# Biofeedback‐Based Therapy in Patients With Myofascial Orofacial Pain and Bruxism: An Umbrella Review

**DOI:** 10.1111/joor.70303

**Published:** 2026-09-09

**Authors:** Celine Beriger, Kathrin Kohout, Aleksandra Zumbrunn, Edwin Ruales‐Carrera, Tan Fırat Eyüboğlu, Mutlu Özcan

**Affiliations:** ^1^ Center for Dental Medicine, Clinic of Masticatory Disorders and Dental Biomaterials University of Zurich Zurich Switzerland; ^2^ School of Dentistry Universidad de Las Américas (UDLA) Quito Ecuador; ^3^ Department of Endodontics, Faculty of Dentistry Istanbul Medipol University Istanbul Türkiye

**Keywords:** biofeedback, clinical trials, myofascial pain, orofacial pain, overview of reviews, umbrella review

## Abstract

**Background:**

Biofeedback therapy is increasingly used to manage myofascial orofacial pain and bruxism, but the overall strength and consistency of the evidence remain unclear.

**Objectives:**

This umbrella review aimed to synthesize and critically evaluate evidence from systematic reviews on the effectiveness of biofeedback therapy for myofascial orofacial pain and bruxism, focusing on pain reduction and masticatory muscle activity.

**Materials and Methods:**

CENTRAL, Embase, LILACS, MEDLINE (PubMed), Scopus, Web of Science, WHO ICTRP, and ClinicalTrials.gov were searched from inception to October 2024 for English‐language systematic reviews. Study selection was performed independently by two reviewers in two stages. Systematic reviews, with or without meta‐analysis, were included. Methodological quality was assessed using AMSTAR‐2, and overlap among reviews was evaluated using the corrected covered area (CCA).

**Results:**

Out of 1670 records identified, 11 systematic reviews met the inclusion criteria. All reported pain‐related outcomes. According to AMSTAR‐2, nine reviews were rated critically low confidence, and two were rated low confidence. Only five reviews assessed the certainty of evidence using GRADE. Moderate overlap among reviews was observed (CCA = 8.48%). Biofeedback consistently reduced masticatory muscle activity, whereas its effects on pain outcomes were inconsistent and limited by heterogeneity across studies.

**Conclusions:**

Evidence supporting biofeedback therapy for myofascial orofacial pain and bruxism remains limited due to methodological weaknesses and heterogeneity. Although biofeedback may reduce masticatory muscle hyperactivity, its effect on pain is uncertain. High‐quality, standardized clinical trials are needed to clarify its therapeutic value.

**Trial Registration:**

Umbrella Review Registration Number: CRD420251005382

## Graphical abstract

This umbrella review of 11 systematic reviews found that biofeedback may reduce masticatory muscle activity, but its effects on myofascial orofacial pain and bruxism remain uncertain. Methodological limitations, heterogeneous protocols, and predominantly low‐quality evidence underscore the need for rigorous, standardized clinical trials.

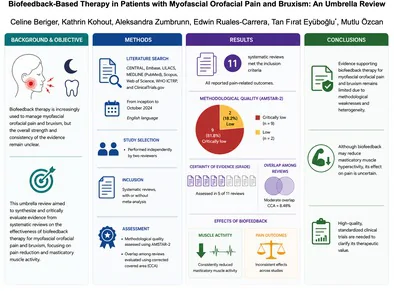

**Abbreviations**
AMSTAR‐2: A MeaSurement Tool to Assess systematic Reviews
BF: biofeedback
CBT: cognitive behavioral therapy
CCA: corrected covered area
CCT: controlled clinical trial
CMD: craniomandibular disorders
DC/TMD: diagnostic criteria for temporomandibular disorders
EMG: electromyography
GRADE: Grading of Recommendations, Assessment, Development and Evaluation
GROOVE: Graphical Representation of Overlap for OVErviews
GSR: galvanic skin response
HRV: heart rate variability
MA: meta‐analysis
nRCT: non‐randomized controlled trial
PICOS: Participants, Intervention, Control, Outcomes and Study design
PRIOR: Preferred Reporting Items for Overviews of Reviews
RCT: randomized controlled trial
RDC/TMD: research diagnostic criteria for temporomandibular disorders
RoB: risk of bias
RSP: respiration‐based feedback
SB: sleep bruxism
SR: systematic review
TMD: temporomandibular disorder
TP: trigger point
VAS: visual analog scale

## 1 Introduction

According to Research Diagnostic Criteria for Temporomandibular Disorders (RDC/TMD) and Diagnostic Criteria for Temporomandibular Disorders (DC/TMD), myofascial pain is classified as a subtype of myalgia. Temporomandibular disorders (TMDs), also referred to as craniomandibular disorders, are commonly associated with functional limitations, including pain during chewing and speaking, and frequently coexist with headaches, migraines, and sleep disturbances [1, 2]. Myofascial pain is characterized by palpation‐induced pain and trigger points (TPs) that may produce localized pain or referred pain beyond the site of palpation. Accordingly, contemporary diagnostic classifications distinguish local myalgia, myofascial pain, and myofascial pain with referral as clinically distinct pain presentations [1, 2].

Patients with painful TMD frequently seek treatment in dental settings. However, no universally accepted standard treatment exists [3]. Symptoms may become chronic over time, and persistent pain can promote maladaptive behavioral and cognitive responses, including protective motor patterns and pain catastrophizing, potentially contributing to greater pain severity and reduced treatment responsiveness [1, 2].

Modern understanding conceptualizes TMD pain within a biopsychosocial framework that recognizes the interaction of biological, psychological, and social factors in symptom development and persistence [4, 5]. Within this model, peripheral nociceptive contributors may include local tissue injury, inflammatory processes, temporomandibular joint pathology, muscle dysfunction, and excessive loading of the masticatory system, whereas central mechanisms involve sensitization and alterations in pain processing that may amplify pain beyond the degree of peripheral tissue involvement [6, 7]. Consequently, persistent pain may reflect central nervous system changes and, in some patients, nociplastic pain features characterized by altered nociceptive processing and pain amplification despite limited evidence of ongoing peripheral tissue pathology, helping to explain why reductions in muscle activity do not necessarily translate into proportional reductions in perceived pain [6, 7]. In addition, psychosocial factors such as anxiety, depression, stress, somatization, catastrophizing, and sleep disturbances have been associated with increased pain intensity, greater functional limitation, and poorer quality of life among patients with TMD [2, 8, 9, 10]. Recent evidence further suggests that oral behaviors, psychological distress, and sleep‐related factors interact with one another and may collectively influence TMD pain rather than acting as isolated risk factors [8, 9].

The aetiology of TMD remains incompletely understood and is generally considered multifactorial [5]. Habits such as gum chewing, bruxism, and nail biting may increase loading of the masticatory system and have been proposed as potential contributors to myofascial pain and TMD [11]. Alqutaibi et al. reported a global TMD prevalence of approximately 29.5%, with myalgia representing the most common diagnosis [12]. Many individuals with myofascial pain report jaw clenching behaviors [13]. Unconscious grinding and clenching during sleep or wakefulness, collectively termed bruxism, is defined as masticatory muscle activity that may also occur in otherwise healthy individuals [14]. Current consensus recognizes awake bruxism and sleep bruxism as distinct behaviors with potentially different underlying mechanisms and clinical implications [14]. Although bruxism may function as a risk factor or clinical sign in some individuals, it is not considered a disorder itself, and studies combining awake bruxism, sleep bruxism, and TMD pain should be interpreted as clinically heterogeneous populations with potentially different treatment targets [14, 15]. Contemporary evidence further suggests that bruxism, pain‐related TMD, and psychosocial factors interact within a broader biopsychosocial framework, in which anxiety, depression, stress, sleep‐related factors, and other oral behaviors may influence symptom presentation and persistence [8, 9, 10, 16].

Reducing bruxism‐related electromyographic activity should therefore not be equated with meaningful pain relief. Muscle activity and pain reduction are related but distinct outcomes, particularly in chronic TMD where central sensitization, overlapping pain conditions, and psychosocial factors may play dominant roles in symptom persistence [7, 15]. Given the multifactorial nature of TMD, contemporary management strategies increasingly favor conservative and interdisciplinary approaches combining self‐care, behavioral interventions, physical therapies, and pharmacological management when indicated [17]. In addition, recognition of comorbid conditions, including inflammatory rheumatic disorders and other systemic contributors, may be important when evaluating patients with TMD because such conditions can influence symptom presentation and management needs [17].

Myalgia related to conditions such as bruxism is therefore best understood within a multidimensional framework in which biological, behavioral, and psychosocial factors influence both symptom presentation and treatment outcomes. Biofeedback has long been recognized as a potentially valuable adjunctive intervention within comprehensive management strategies [18]. It facilitates self‐regulation of physiological processes through real‐time monitoring of biological signals such as muscle tension, skin temperature, heart rate variability, or skin conductance. These signals are presented through visual or auditory feedback, allowing patients to develop voluntary control over physiological responses associated with muscle tension, stress, and autonomic regulation under the guidance of trained clinicians [19, 20].

Although several reviews have evaluated biofeedback for orofacial pain and bruxism, important uncertainties remain regarding the interpretation and clinical applicability of the available evidence. First, reductions in masticatory muscle activity appear more consistent than improvements in pain outcomes, raising questions about whether neuromuscular changes translate into clinically meaningful pain reduction [6, 7, 14, 15]. This issue is particularly relevant because TMD pain is now understood as a multifactorial condition in which peripheral, central, and psychosocial mechanisms may contribute differently across patient populations [4, 5, 6, 7, 8, 9, 10, 16]. Second, TMD pain encompasses heterogeneous clinical presentations with varying chronicity, psychosocial characteristics, and contributing mechanisms, making it unclear whether biofeedback effectiveness differs across patient subgroups [1, 2, 6, 7, 15]. Third, methodological variability across reviews, including differences in eligibility criteria, intervention definitions (particularly the evaluation of biofeedback alone or in combination with other therapeutic approaches), outcome measures, evidence synthesis methods, certainty assessment, and overlap of included primary studies, may influence the consistency and interpretation of reported findings and complicate comparison across reviews [21, 22, 23]. Consequently, repeated findings across reviews should not necessarily be interpreted as independent confirmation of effectiveness.

There is currently no integrated evaluation capable of simultaneously examining whether reductions in masticatory muscle activity are associated with clinically meaningful reductions in pain, whether treatment effectiveness differs across TMD‐related pain phenotypes, and to what extent conclusions are influenced by methodological quality and overlap among primary studies. An umbrella review is uniquely suited to address these unresolved questions because it permits simultaneous assessment of evidence consistency, methodological quality, and primary‐study redundancy [21, 22, 23, 24].

This umbrella review aimed to synthesize systematic reviews and meta‐analyses, assess their quality, and evaluate consistency in findings. It focused on the effectiveness of biofeedback interventions for myofascial and orofacial pain, identified research gaps, and offered recommendations. Specifically, it examined whether reductions in masticatory muscle activity are consistently linked to pain reduction, whether the available evidence allows phenotype‐specific interpretation of treatment effects, and which methodological limitations, including overlap among primary studies, may restrict the clinical applicability of current evidence.

## 2 Materials and Methods

This umbrella review followed the guidelines of the Preferred Reporting Items for Overviews of Reviews (PRIOR) and was registered with the National Institute for Health Research PROSPERO, the International Prospective Register of Systematic Reviews [23]. The registration is publicly available under the number CRD420251005382. Ethics approval was not required for this overview.

### 2.1 Information Sources and Search Strategy

With the assistance of an experienced health sciences librarian, a comprehensive search of the following electronic databases was conducted on November 26th, 2024, to identify relevant studies, including CENTRAL, Embase, LILACS, MEDLINE (PubMed), Scopus, Web of Science, WHO ICTRP, and ClinicalTrials.gov. All available studies on biofeedback methods were considered, including the earliest published reviews in this field. The search covered studies published from September 1972 to October 2024 and was restricted to English‐language articles only. No additional articles were identified through manual searches. The restriction to English‐language publications was applied for feasibility reasons; however, the comprehensive database coverage aimed to minimize potential language bias. The search was re‐run prior to final analysis to ensure that no additional eligible reviews had been published after the initial search date. The search strategy for each database is provided in Appendix S1.

### 2.2 Study Selection and Eligibility Criteria

The eligibility criteria were defined using the PICOS framework (Population, Intervention, Comparison, Outcomes, and Study Design) [25]:

Population (P): Adults with myofascial orofacial pain and adults with myofascial orofacial pain and bruxism.

Intervention (I): Biofeedback.

Comparison (C): Any other type of intervention.

Outcomes (O): Primary: pain perception; Secondary: masticatory muscle activity.

Study design (S): Systematic reviews (SR) and meta‐analysis (MA).The exclusion criteria were as follows: 1—Articles in languages other than English; 2—Studies without a detailed description of methods and outcomes; 3—Studies including participants under the age of 18 years; 4—Patients with acute pain originating from components of the masticatory system other than the muscles (e.g., caries); 5—Publication types other than SRs.

### 2.3 Selection Process

The identified articles were exported to a reference manager (EndNote 21, Clarivate, Philadelphia, PA), and duplicates were removed. For screening, all identified references were organized in a Microsoft Excel spreadsheet. Two independent reviewers (C.B. and K.K.) performed study selection in a two‐step process: first, titles and abstracts were screened to exclude clearly irrelevant records, followed by full‐text assessment of potentially eligible articles. Any disagreement between the reviewers at either stage was discussed and resolved in consultation with a third reviewer (M.Ö.). A quantitative synthesis was planned if the included systematic reviews provided sufficient and comparable data, such as effect estimates presented in forest plots.

### 2.4 Data Collection Process

Data extraction was performed using a structured Excel spreadsheet to ensure accuracy and consistency. Extracted data included the following:

- Study characteristics (authors, year of publication, country, objectives, primary studies, and journal impact factor).
- Participants and diagnostic details (population and diagnostic method).
- Interventions and outcomes (interventions and comparators, outcome(s), follow‐up range, results, and conclusion).
- Review method and synthesis (Cochrane review, Grading of Recommendations, Assessment, Development, and Evaluation (GRADE), MA, Risk of Bias (RoB) assessment tool, software used in the MA, number of studies included in the MA).
- Reporting and search details (protocol registration, database(s) searched, grey literature, experts, conflict of interest).

If required, authors were contacted to clarify missing data.

### 2.5 Assessment of Methodological Quality

Methodological quality of included SRs was assessed using the AMSTAR‐2 (A MeaSurement Tool to Assess systematic Reviews) checklist [24]. The AMSTAR‐2 consists of 16 items. Each item was rated as “yes”, “partial yes”, or “no”. Based on the pattern of responses, an overall confidence level of “high”, “moderate”, “low”, or “critically low” was assigned to each review, following the criteria proposed by Shea et al. [24].

### 2.6 Synthesis of Results

Evidence from the SRs was synthesized using a narrative approach. Results were presented in tables, figures, and color charts. To address potential data overlap from duplicate primary studies across reviews, the degree of overlap was assessed using the Corrected Covered Area (CCA) and further illustrated with the Graphical Representation of Overlap for OVErviews (GROOVE) tool [26]. CCA values were interpreted according to established thresholds (0%–5% slight overlap; 6%–10% moderate overlap; 11%–15% high overlap; > 15% very high overlap).

### 2.7 Certainty Assessment

For each included SR, the certainty of the evidence was reported, when available, using the GRADE approach [26]. When GRADE assessments were available, information on certainty ratings and reasons for downgrading was extracted. SRs without GRADE application were included in the narrative synthesis but were not considered in the certainty assessment. No independent reassessment of certainty at the umbrella review level was performed, as the objective was to synthesize and report the certainty ratings provided within the included systematic reviews.

## 3 Results

### 3.1 Study Selection

A total of 1670 records were identified through database searches across multiple platforms, as outlined in the search strategy. After removing duplicates and retracted records, 777 articles remained for further evaluation. Studies identified as potentially relevant (*N* = 56) underwent full‐text review. Each full text was assessed against the inclusion and exclusion criteria established in the review protocol. Eleven SRs met the inclusion criteria and were included in the umbrella review. Three of these reviews were considered for quantitative synthesis. However, the extracted data were insufficient to perform a meta‐analysis. Consequently, findings from all included reviews were summarized narratively. Disagreements during the screening process were resolved through discussion, with arbitration by a third independent reviewer (M.Ö.). Inter‐reviewer agreement during screening, as measured by Cohen's kappa, was recorded to ensure reliability. Cohen's kappa scores for agreement between reviewers ranged from 0.89 to 1.00. Reasons for excluding SRs are provided in Table S1 (Appendix S2). Figure 1 illustrates the study identification and selection process.

**FIGURE 1:**
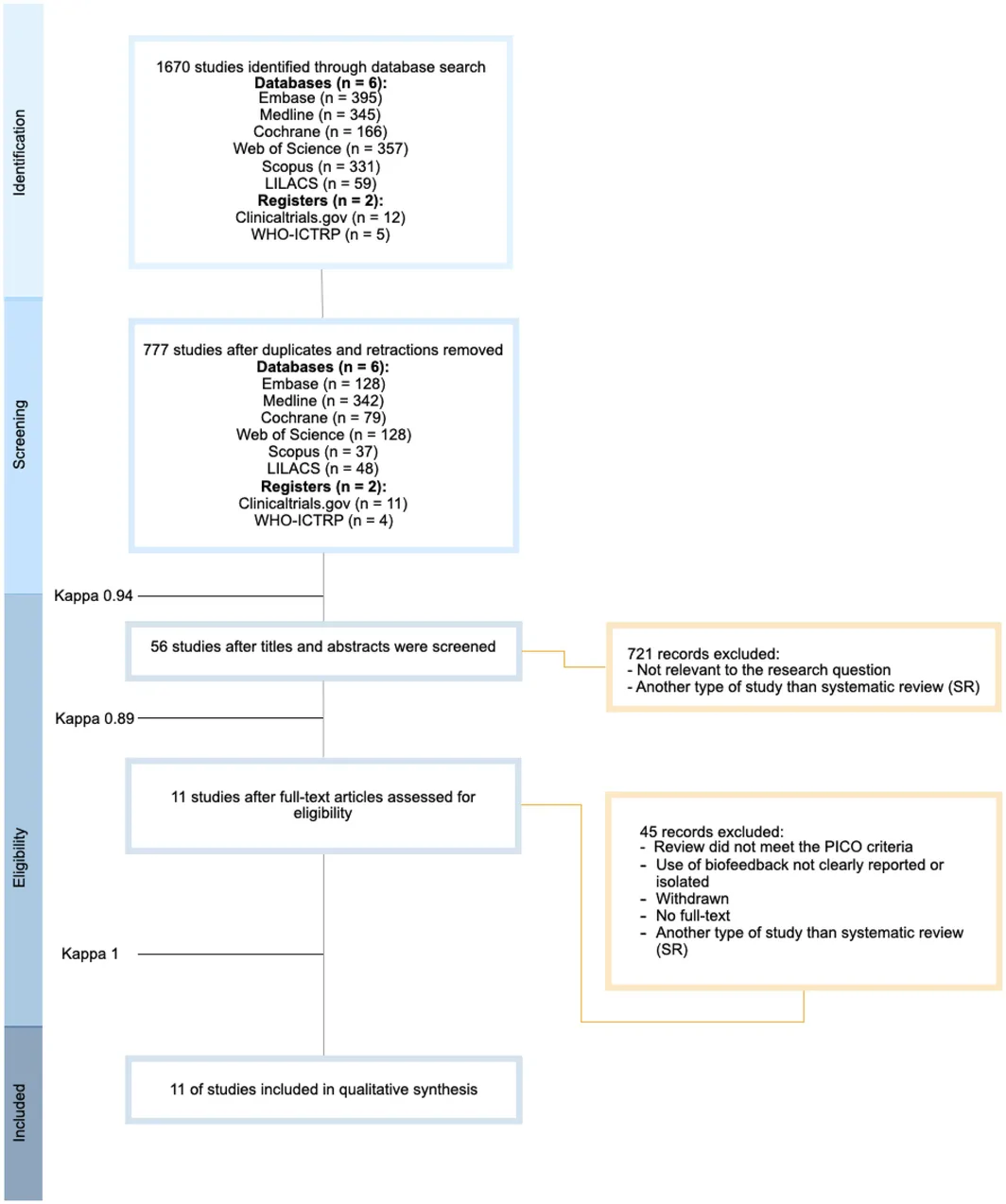
Flow diagram illustrating the study selection process across phases of identification, screening, eligibility, and inclusion, adapted for overviews of reviews in line with the PRIOR statement, Gates et al. [23].

### 3.2 Characteristics of Systematic Reviews

The included SRs were published between 2003 and 2023 in ten journals. All SRs were published in English. The reviews were conducted in several countries: Canada, China, USA, Colombia, Norway, Singapore, and Switzerland [27], Romania [28], Poland [29], UK [30], Lithuania [31], Japan [32], Brazil [33], China [34], Canada [35, 36], and Sweden [37]. Detailed PICOS characteristics are provided in Table S2 (Appendix S2), while a descriptive overview of the included systematic reviews is presented in Table 1.

**TABLE 1:** Descriptive overview of included systematic reviews (*n* = 11).

Author (year)	Objective(s)	Results	Conclusions
			Type of biofeedback
Aggarwal et al. (2019) [30]	“To determine whether, in adults with chronic orofacial pain including temporomandibular disorders (TMD), self‐management interventions more effective than usual care in improving long‐term outcomes related to pain intensity and psychosocial well‐being; whether the biofeedback component of interventions shows an additional treatment effect compared to no biofeedback; the effectiveness of self‐management for subtypes of chronic orofacial pain in particular TMD which is the most common subtype”	**Self‐management versus usual care:** **Pain (short term):** *I* ^2^ = 62% (results could not be pooled), nine studies reported relevant data **Pain (long term):** *I* ^2^ = 7%, SMD −0.32, (95% CI: −0.47 to −0.17), (results could be pooled, significant difference favoring self‐management, nine studies reported relevant data) When analysing the different subgroups of interventions separately, statistically significant effects were found: Biofeedback combined with CBT showed a stronger effect than self‐care CBT alone (as shown below: subgroup CBT and biofeedback) **Muscle palpation pain** *I* ^2^ = 63%, SMD −0.58, (95% CI −0.92 to −0.24), three studies reported relevant data, evidence was insufficient to assess the long‐term effects of individual interventions on muscle palpation pain. **Activity interference** *I* ^2^ = 79%, SMD −0.29, (95% CI −0.47 to −0.11), eight studies reported relevant data **Depression:** *I* ^2^ = 0%, SMD −0.32, (95% CI −0.50 to −0.15), significant difference favoring psychosocial interventions, seven studies reported relevant data Self‐care CBT and CBT/biofeedback each showed a significant benefit over usual care in terms of depression **Subgroup self‐care CBT** **Pain (long term):** SMD −0.26, (95% CI −0.45 to −0.07) **Activity interference** SMD −0.37, (95% CI −0.57, −0.16) **Depression:** SMD −0.27, (95% CI −0.49 to −0.05) **Subgroup CBT and biofeedback:** **Pain (long term):** SMD −0.46, (95% CI −0.72 to −0.20) **Depression:** SMD −0.41, (95% CI −0.68 to −0.13) **Meta‐regression:** Biofeedback on long‐term pain: five out of the eleven studies with long term pain as an outcome also used biofeedback, SMD −0.16, (95% CI −0.48 to 0.17, *p*‐value = 0.360) **Depression:** Three out of the eight studies with depression as an outcome also used biofeedback (−0.13, 95% CI −0.50 to 0.23, *p*‐value = 0.475)	1. Strong evidence supporting self‐management strategies to improve long‐term outcomes in patients with chronic orofacial pain and TMD No additional benefit from biofeedback alone was observed. A meta‐regression was possible for the six studies that included biofeedback. The analysis indicated that biofeedback alone had no significant effect, with residual heterogeneity being very low (*I* ^2^ = 7%) 2. CES, audio feedback, visual feedback, CBT with biofeedback
Jokubauskas and Baltrušaitytė (2018) [31]	“What is the effect of biofeedback on SB activity and other patient‐related variables, compared with other treatment options, in patients with SB?”	**Pain** No consistent reduction in clinical pain levels was observed **Bruxism‐related EMG activity** Two additional RCTs by Jadidi et al. were included to allow pooled analysis; due to methodological and reporting heterogeneity, EMG episodes/hour after the first night showed a near‐significant difference between CES and control (md = −5.05, 95% CI = −10.71 to 0.62), after five nights, a significant reduction in favor of CES was found (md = −7.18, 95% CI = −12.54 to −1.83)	1. This SR is an update of the 2014 review by wang et al. and provides a current synthesis of the literature on biofeedback‐based management of SB, Only three RCTs were included in the quantitative synthesis due to differences between studies; two of them came from an earlier review.; GRADE assessment: “moderate degree of certainty that one of the biofeedback modalities, CES, shows a significant effect on the reduction in SB‐related EMG episodes after a short‐term treatment period compared with the no‐treatment group. However, evidence of long‐term effects is lacking”; qualitative synthesis did not show a reduction in clinical pain levels; each study demonstrated a notable decrease in EMG activity associated with sleep bruxism after the application of cranial electrotherapy stimulation (CES); the results leave it unclear whether CES has only short‐term effects or if longer treatment could enhance and prolong its impact; although many studies report positive outcomes, the evidence remains limited due to the small number of studies included in the meta‐analysis 2. CES, audio feedback, vibratory feedback, EMG‐triggered electrical stimulation biofeedback
Wang et al. (2014) [34]	“To evaluate the efficacy of any biofeedback treatment on SB”	**SB episodes (EMG measured):** (*I* ^2^ = 61%, *p* = 0.11), md = −4.47, (95% CI = −12.33, 3.38), three studies reported relevant data (SB episodes between CES and control) EMG SB episodes decreased significantly after nocturnal biofeedback and splint therapy **Bruxism‐related EMG activity** Five studies reported relevant data, no meta‐analysis performed, audio feedback superior to control, difference between audio feedback and stress‐reduction behavioral counselling was not significant, after two weeks of treatment and at six months follow‐up, audio biofeedback led to about a 70% reduction in EMG activity over three months, without between‐group comparisons EMG activity decreased significantly after nocturnal biofeedback and splint therapy, not after diurnal biofeedback, massed negative practice or control Visual biofeedback and TENS decreased EMG activity (TENS higher EMG levels after treatment) **Pain** Only one study reported relevant data, no significant changes among the baseline (CES and control)	1. Substantial variability and inconsistency in biofeedback modalities, control conditions, and outcome measures, limited number of RCTs with the same interventions and outcome indexes (five of the seven studies, data could not be pooled and were therefore only described qualitatively); EMG activity decreased after six weeks of CES, while no effect was seen after a single night. Longer CES treatment may be needed for measurable effects 2. CES, audio feedback, visual EMG feedback, diurnal feedback
Yao et al. (2023) [27]	“We explored the comparative effectiveness of available therapies for chronic pain associated with temporomandibular disorders (TMD)”	**Pain relief** The three most likely effective therapies were (RD for achieving pain relief of 1 cm on a 10 cm scale): CBT augmented with biofeedback or relaxation therapy: 36% (95% CI = 33–39); therapist‐assisted jaw mobilization: 36 (95% CI = 31–40) and manual trigger point therapy (95% CI = 29–34) Certainty of effects low to very low: Biofeedback: md = −1.03 (95% CI = −2.42 to 0.37) **Physical functioning** The certainty of effects on physical functioning for other interventions, including biofeedback, was low or very low. Biofeedback: md = 6.45 (95% CI = −31.28 to 44.19) **Adverse events** Biofeedback: No results	1. Restricted only to moderate or high certainty evidence, interventions with coping and encourage movement and activity are most effective for reducing chronic TMD pain (augmented CBT). “Several interventions commonly in use for chronic TMD pain are supported by only low or very low certainty evidence” (biofeedback alone) 2. Computer‐controlled auditory tone and pulsating feedback; biofeedback and CBT combined; A J&J Model M‐57 EMG Biofeedback Unit and a J&J Model T‐68 Temperature Biofeedback Unit; BiotrainerTM visual biofeedback software
Miron et al. (2022) [28]	“[…] to conduct a systematic review of the therapeutic approaches of nocturnal bruxism, including only randomized controlled clinical trials published in the scientific literature”	**Pain** Reduction in facial muscle pain	1. Conclusions should be drawn with caution, significant reduction in bruxism events, reduction in facial muscle pain 2. BruXane splint (vibratory and auditory feedback)
Florjanski et al. (2019) [29]	“[…] to review the available modern literature to determine the effects and efficiency of masticatory muscle activity management based on biofeedback”	**Masticatory muscle activity** Significant correlation between biofeedback and muscle activity reduction; in two studies with follow‐up, EMG activity returned to baseline, and in Conti et al. (2014) [38] was no longer significantly different from the control **Pain** Impact of biofeedback unclear, different results (Criado et al. 2016) [39]: significant decrease of symptoms; Raphael et al. (2013) [40]: decrease of pain also after follow‐up; Conti et al. (2014) [38]: no change in comparison to baseline levels; Jadidi et al. (2008) [41] no statistically significant change in comparison to baseline levels	1. Quality of evidence low to very low, sample sizes in included studies small, impact of biofeedback on pain unclear due to a wide variety of symptoms which makes comparison difficult. Biofeedback useful in decreasing muscle activity 2. Visual feedback, vibratory feedback, audio feedback, CES
Iwasaki et al. (2018) [32]	“[…] to clarify the usefulness of CBT for TMD and provide updated relevant information”	**Pain** CBT augmented with biofeedback significantly reduced chronic pain levels compared to nonintervention	1. Efficacy of CBT could not be clarified, biofeedback only in combination with CBT but is considered as the most effective for reducing pain perception in two included studies 2. EMG biofeedback unit, temperature biofeedback unit
Amorim et al. (2018) [33]	“[…] to examine the effect of physical therapy in bruxism treatment”	**Pain** TENS shows no higher effect than visual BF, but it improves muscle pain and generated muscle relaxation similar to visual BF Studies using GrindCare reported inconsistent findings, with muscle relaxation leading to pain relief in some cases but not in others **EMG events, bruxism events** BF statistically decreases in bruxism events duration, decrease in tonic EMG events **Muscle activity** Five studies using GrindCare show a decrease in muscle activity	1. Among electrotherapy, GrindCare (BF) appeared to have the greatest improvement in muscle activity; the evidence (27) was very low quality for all studies 2. CES, auditory, visual and vibratory feedback
Medlicott and Harris (2006) [35]	To examine the effectiveness of physical therapy interventions for TMD	Therapies with relaxation techniques and biofeedback may be more effective than placebo treatment or occlusal splints (implication for clinical practice) The following outcome variables were used: pain to palpation, pain (3‐point scale), pain intensity (numerical or VAS) Considerable heterogeneity was observed in biofeedback interventions, making comparisons difficult	1. Inclusion criteria were not identified in seven out of 30 studies and varied among the included studies. Subjects with myofascial TMD were included in 60% of the studies; “a variety of interventions were used to treat the 3 TMD subgroups in the first axis. Interventions were grouped into 1 of 3 areas: exercise, electrotherapy, and biofeedback. Within the 3 areas, the interventions were often heterogeneous, making comparisons difficult. The use of multiple interventions in a number of studies resulted in recommendations based on a multi‐intervention program because the effective ness of a single intervention alone was not examined.” Implications should be considered with caution Review has several limitations, only english articles are included 2. EMG biofeedback or not reported. For more information see Supporting Information S5
McNeely et al. (2006) [36]	“to assess the evidence concerning the effectiveness of physical therapy interventions in the management of temporomandibular disorders”	**Pain** TENS treatment not significantly better in improving pain than biofeedback for myogenous TMD (one weak study) Biofeedback not significantly better than relaxation therapy or occlusal splint therapy (two weak studies) Substantial heterogeneity was present among the studies regarding TMD type, interventions, comparators, and treatment schedules **Oral opening** Biofeedback significant improvement compared to occlusal splint	1. Studies examining biofeedback were considered weak, numerous concerns with the design of the primary studies. No evidence was identified indicating that any electrophysical modality is effective in reducing TMD pain 2. Not reported (see Supporting Information S5)
Jedel and Carlsson (2003) [37]	“to assess the efficacy of biofeedback, acupuncture and transcutaneous electric nerve stimulation in the management of temporomandibular disorders”	**Pain** Biofeedback: No evidence of efficacy of biofeedback in the management of TMD. Biofeedback alone shows indifferent results CBT combined with biofeedback shows a positive result (1 CCT). The following outcome variables were used: CPI, GCPS, POMS. Biofeedback in combination showed a statistically significant reduction in the CPI after treatment (*p* = 0.000) and differed significantly from the untreated control group (*p* = 0.014). No significant change was observed in the GCPS. Overall, biofeedback in combination demonstrated statistically significant improvement after treatment (*p* < 0.001)	1. No evidence of efficacy of biofeedback in the management of TMD. All studies are of low quality 2. Not reported (see Supporting Information S5)

Abbreviations: CPI, characteristic pain index; GCPS, graded chronic pain; POMS, profile of mood states; TENS, transcutaneous electrical nerve stimulation; TMD, temporomandibular dysfunction.

### 3.3 Bibliometric Characteristics

Details of the bibliometric characteristics are provided in Table S3 (Appendix S2).

### 3.4 Methodological Quality

Of the eleven included SRs, nine were rated as having “critically low” confidence, while two were rated “low” [27, 30]. The most critical domains were the protocol, adequacy of the literature search, justification for excluding individual studies, risk of bias assessment, quantitative combination of results, and consideration of bias in interpretation. Quantitative analysis was conducted in five systematic reviews [27, 30, 31, 32, 34], and publication bias was assessed and its potential impact discussed in two of them [27, 30]. Figure 2 provides further details on the evaluation of methodological quality.

**FIGURE 2:**
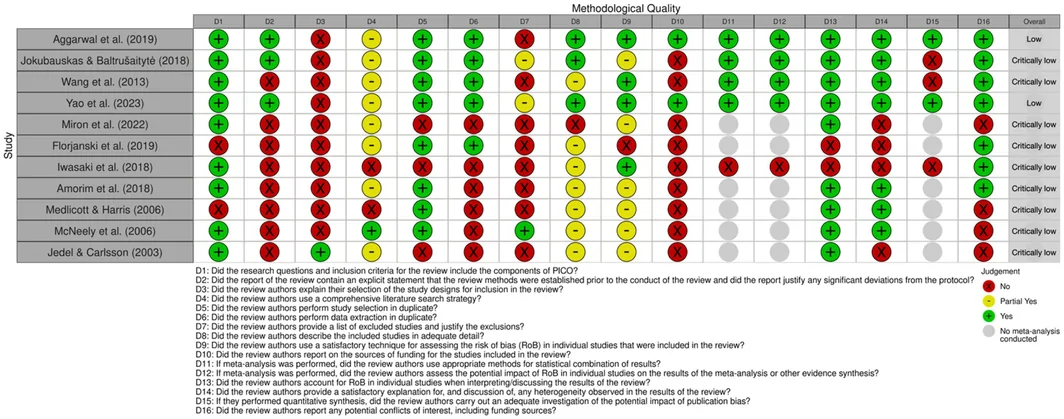
Methodological quality was assessed using the AMSTAR‐2 tool (14) and presented graphically as a “traffic light” plot. The figure was generated with robvis (visualization tool), with green indicating a “yes” response, yellow a “partially yes” response, red a “no” response, and blank cells indicating no meta‐analysis (16–26).

### 3.5 Primary Study Overlap

The SRs collectively included 33 primary studies relevant to the PICO criteria. Analysis revealed a moderate overlap among the SRs, corresponding to a CCA of 8.48%. Figure 3 presents the GROOVE illustrating these overlaps, while Table S4 (Appendix S2) provides a comprehensive evidence matrix showing which primary studies are represented in each review.

**FIGURE 3:**
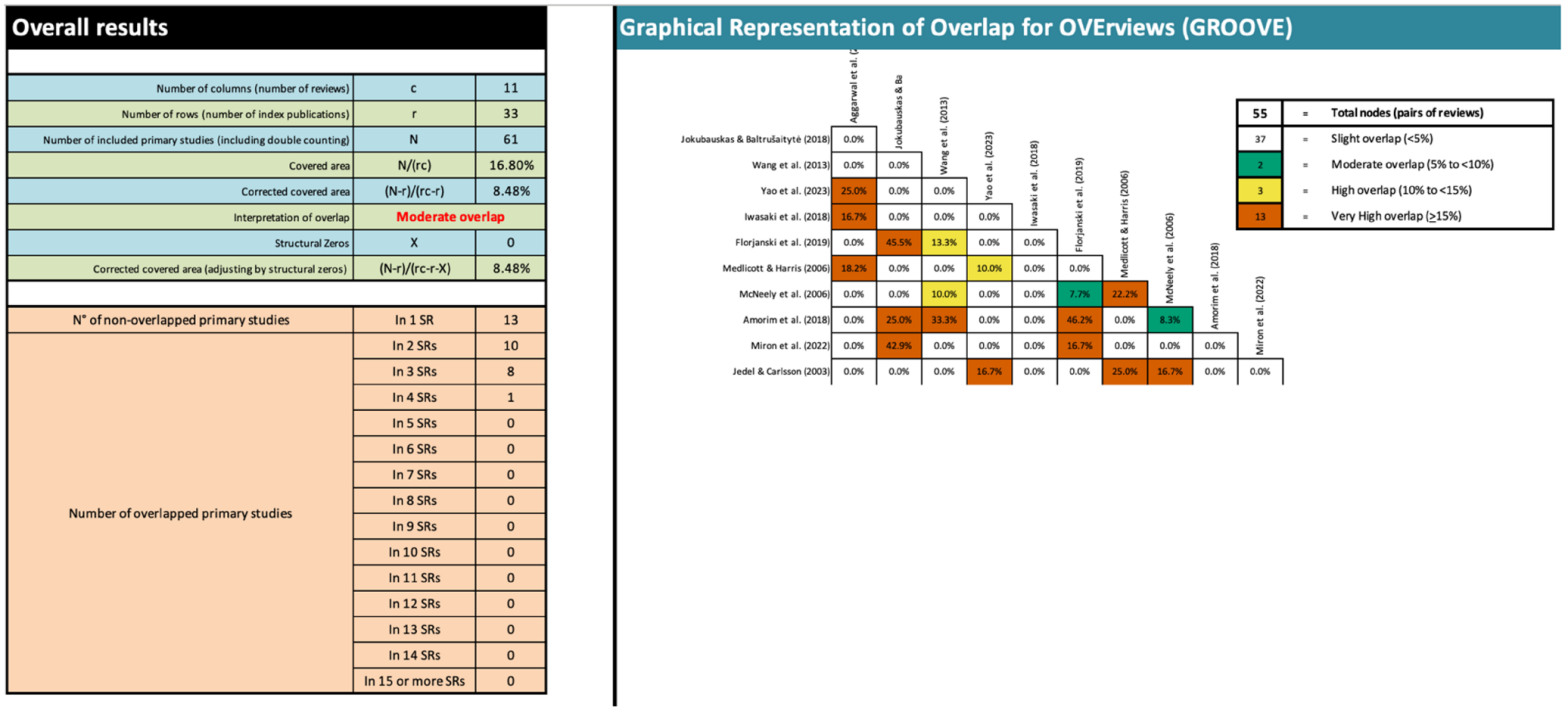
Graphical Representation of overlap for OVErviews (GROOVE). Figure generated with Microsoft Excel (16–26).

### 3.6 Synthesis of Results of Systematic Reviews

Table S5 (Appendix S2) summarizes the specific characteristics of biofeedback interventions across the included systematic reviews. Table 2 summarizes the effects of biofeedback interventions on pain and muscle activity.

**TABLE 2:** Effect of treatments according to primary and secondary outcomes.

		Primary outcomes				Secondary outcomes			
		Pain				Muscle activity			
Treatment	Author(s), year	Posititve	Negative	Neutral	Inconclusive	Positive	Negative	Neutral	Inconclusive
Biofeedback	Aggarwal et al. (2019) [30]			✓ Long‐term pain relief					
	Jokubauskas and Baltrušaitytė (2018) [31]				✓ Pain reduction	✓ EMG activity			
	Wang et al. (2014) [34]			✓ Pain reduction		✓ EMG activity			
	Yao et al. (2023) [27]				✓ Pain relief				
	Miron et al. (2022) [28]	✓ Pain reduction							
	Florjanski et al. (2019) [29]				✓ Pain reduction, Pain intensity	✓ Masticatory muscle activity			
	Amorim et al. (2018) [33]				✓ Pain reduction	✓ Masticatory muscle activity			
	Medlicott and Harris (2006) [35]				✓ Pain reduction				
	McNeely et al. (2006) [36]				✓ Pain reduction				
	Jedel and Carlsson (2003) [37]				✓ Pain reductioni				
CBT augmented with biofeedback	Yao et al. (2023) [27]	✓ Pain relief							
	Aggarwal et al. (2018) [30]	✓ Long‐term pain relief							
	Iwasaki et al. (2018) [32]	✓ Chronic pain							

*Note:* Light blue identifies biofeedback interventions, while darker blue identifies cognitive behavioral therapy (CBT) augmented with biofeedback. Light green identifies the included systematic reviews. Bright green indicates outcomes for which a positive treatment effect was reported. Dark grey indicates that the corresponding outcome was not assessed or reported. Unshaded cells represent outcome categories in which no result was recorded; where text appears in an unshaded cell, it indicates the corresponding neutral or inconclusive finding specified by the column heading.

### 3.7 Pain

Jedel and Carlsson reviewed three controlled clinical trials (CCTs), one of which combined biofeedback with cognitive behavioral therapy (CBT) [37]. The SR did not provide a separate analysis of this intervention [37]. Comparators were splint therapy and untreated controls [37]. No evidence supported the efficacy of biofeedback for TMD pain [37]. McNeely et al. assessed four randomized controlled trials (RCTs) using biofeedback as an intervention [36]. Regarding pain, biofeedback was not more effective than TENS for myogenous TMD and showed no advantage over relaxation therapy or occlusal splints [36]. Considerable heterogeneity existed among studies in TMD type, interventions, comparators, and treatment schedules [36]. There is no evidence supporting biofeedback to reduce TMD pain [36]. On the other hand, Medlicott and Harris reviewed seven RCTs [35]. One review was rated as having “moderate” methodological quality, while the others were considered “weak” [35]. Considerable heterogeneity was observed in the biofeedback interventions, making direct comparisons between studies difficult [35]. Despite the limitations of the SR, the authors suggested that interventions combining relaxation techniques and biofeedback may offer greater benefits than placebo or occlusal splint treatments [35].

One RCT assessing pain as an outcome was included in Wang et al.'s study [34]. CES was used as the biofeedback intervention [34]. Pain was measured using the number of painful muscles, characteristic pain intensity, and the McGill Pain Questionnaire [34]. No changes in pain were observed between pre‐ and post‐treatment. The RoB for this study was rated as moderate [34].

Amorim et al. reviewed twelve clinical trials, eight of which were RCTs [33]. Comparators included various control conditions [33]. Visual biofeedback was associated with reduced muscle pain, similar to TENS and MENS [33]. GrindCare studies showed inconsistent results, with pain improvement in some cases and no association with muscle relaxation in others [33]. Study quality was assessed using the PEDro and Jadad scales, and most biofeedback trials were rated as having a high risk of bias [33]. Two studies achieved Jadad scores of 3 and 4 [33]. Iwasaki et al. reviewed one RCT and found that CBT combined with biofeedback led to greater reductions in chronic pain than no intervention at one‐year follow‐up [32]. The outcome referred to the combined use of both methods rather than biofeedback alone. According to the authors, RoB was assessed with the Cochrane tool [32]. For the included RCT, random sequence generation, blinding of outcome assessment, allocation concealment, and other sources of bias were rated as unclear. Incomplete outcome data and selective reporting were judged as low risk [32].

Jokubauskas and Baltrušaitytė reviewed four RCTs and two non‐randomized controlled trials (nRCTs) [31]. Comparators were various treatments currently used for SB [31]. The authors noted that three studies assessed the effect of CES on pain [31]. Overall, the findings did not support a consistent pain‐reducing effect of CES [31]. One study reported significant reductions in palpation‐induced and spontaneous pain [31]. Methodological quality was rated moderate to high for the four RCTs and low for the two nRCTs [31]. In another study, Aggarwal et al. assessed six RCTs relevant to biofeedback [30]. In all trials, control groups were similar and received standard care, including conservative measures such as education, counselling, and an intraoral flat‐plane appliance [30]. Self‐management interventions, including biofeedback, were examined in four of the nine studies addressing long‐term pain [30]. Low heterogeneity (*I* ^2^ = 7%) allowed pooling of the results, showing that these interventions led to an estimated 16% reduction in long‐term pain compared with standard care (mean difference = −0.32, 95% CI: −0.47 to −0.17) [30]. Separate analysis of the intervention subgroups revealed significant effects, with biofeedback combined with CBT (mean difference = −0.46, 95% CI: −0.72 to −0.20) showing a larger impact than self‐care CBT (mean difference = −0.26, 95% CI: −0.45 to −0.07) [30]. Long‐term muscle palpation pain was reported in three studies, two of which included biofeedback. However, due to high heterogeneity (*I* ^2^ = 63%) and limited data, no reliable conclusions could be drawn [30]. The review confirms that self‐management interventions improve long‐term outcomes in chronic orofacial pain and TMD, as all included studies were at low risk of bias [30]. However, the authors highlight that biofeedback provides no clear additional benefit and should be further evaluated given the extra equipment, training, and patient time required [30].

In 2019, Florjanski et al. reviewed ten studies, four of which examined pain as an outcome [29]. The evidence regarding the impact of biofeedback on pain was inconsistent [29]. Some studies reported improvements in muscle pain, jaw movement pain, or self‐reported pain [29]. Others found no relevant changes in pain intensity or pressure pain threshold [29]. The authors noted that the heterogeneity of symptoms and protocols made comparisons difficult and that the impact of biofeedback on pain remains inconclusive [29].

All nine studies reviewed by Miron et al. were rated good quality, although only one RCT evaluated a biofeedback intervention [28]. Biofeedback was associated with reductions in facial muscle pain compared with an occlusal splint [28]. Results were limited by small sample sizes and variable bruxism symptoms [28]. The authors noted that longer treatment periods may be necessary to achieve the full effect, as biofeedback relies on active patient participation rather than passive devices, and that conclusions should be drawn with caution [28].

Furthermore, Yao et al. recently evaluated three RCTs involving biofeedback [27]. Two trials assessed biofeedback alone, while one study included both biofeedback alone and biofeedback combined with CBT [27]. All three studies were included in the network meta‐analysis [27]. Risk of bias was high in two studies and probably low in one [27]. For pain relief, biofeedback alone showed low to very low‐certainty evidence and limited effectiveness (mean difference = −1.03, 95% CI = −2.42 to 0.37). No data were available on the proportion of patients achieving a clinically meaningful improvement [27]. In contrast, CBT combined with biofeedback demonstrated moderate to high‐certainty evidence and ranked among the most effective interventions (mean difference = −2.62, 95% CI = −3.03 to −2.20), with 36% of patients achieving at least a 1 cm reduction in pain (risk difference ≥ minimal important difference = 36%, 95% CI = 33–39) [27].

Overall, evidence that biofeedback alone reduces pain in TMD is limited and inconsistent. Several studies [27, 34, 36, 37] found no significant benefit over standard care, relaxation therapy, TENS, or occlusal splints. Some trials [28, 29, 33] reported reductions in muscle or facial pain, but results were heterogeneous and study quality varied. Biofeedback combined with CBT showed more consistent and clinically relevant pain reductions in several studies [27, 30, 32]. Study heterogeneity, small sample sizes, and variable methodological quality limited comparability, and the effect of biofeedback alone remains uncertain.

### 3.8 Muscle Activity

In the SR by Wang et al., relevant data were reported in five studies [34]. Audio biofeedback showed the most consistent effects [34]. A clear advantage over control was reported, while no difference from stress‐reduction counselling was detected [34]. In one study, EMG activity decreased by about 70% after three months of audio feedback, and the effect persisted at six months. Significant decreases were also observed with nocturnal audio biofeedback and visual biofeedback [34]. In contrast, diurnal biofeedback did not lower EMG activity [34]. RoB assessment indicated that two studies had moderate risk of bias, while three were rated as high risk [34].

Amorim et al. reported that biofeedback reduced muscle activity across five primary studies [33]. They further noted that GrindCare (BF) appeared to produce the most pronounced improvement in muscle performance among electrophysiological interventions [33]. However, the authors emphasized that most studies were methodologically weak, resulting in very low‐certainty evidence [33]. Jokubauskas and Baltrušaitytė assessed six primary studies and conducted a meta‐analysis that included one of these along with two additional RCTs [31]. The pooled analysis of the first night showed a near‐significant difference between CES and control (mean difference = −5.05, 95% CI: −10.71 to 0.62) [31]. A significant effect was observed when outcomes from the fifth night of two studies were combined (mean difference = −7.18, 95% CI: −12.54 to −1.83) [31]. According to the qualitative assessment by Jokubauskas and Baltrušaitytė, CES reduced SB‐related EMG activity in all included studies [31]. Treatment periods ranged from two days to six weeks [31]. In some studies, the effect lasted more than five days, while in others EMG activity returned to baseline within two weeks, with reductions generally larger during the first nights of follow‐up [31]. However, the authors noted uncertainty about the duration of this effect and cautioned against interpreting the results [31].

Florjanski et al. reviewed 10 studies, which were divided into two groups based on intervention type: biofeedback (including audio, visual, and vibratory feedback) and CES [29]. Most studies suggested a significant association between biofeedback and reduced masticatory muscle activity [29]. However, the authors stressed that these findings should be viewed with caution due to small sample sizes, heterogeneous protocols, and the lack of long‐term follow‐up [29].

Overall, the included reviews indicate that biofeedback‐based interventions can reduce masticatory muscle EMG activity. The magnitude and persistence of these effects varied across studies, and the certainty of the evidence remains low due to small samples and methodological limitations.

### 3.9 Certainty Assessment

Of the eleven included SRs, five assessed the certainty of evidence using the GRADE approach [42]. Yao et al. assessed the certainty of evidence for pain relief with biofeedback alone and CBT combined with biofeedback [27]. Pain relief was rated “moderate” for augmented CBT and “low” for biofeedback alone [27]. The authors concluded, based on moderate‐certainty evidence from participants, that CBT combined with biofeedback is among the most effective approaches for managing pain in patients with chronic TMD compared with placebo or sham procedures [27]. Aggarwal et al. assessed the certainty of evidence for the intervention CBT augmented with biofeedback [30]. Certainty ratings were provided for five outcomes [30]. Short‐term and long‐term pain were both rated “high” [30]. No GRADE assessment was conducted for biofeedback alone or for the outcome muscle activity [30]. In the SR by Jokubauskas and Baltrušaitytė, the GRADE approach was applied to the intervention biofeedback (CES), and the outcome examined was EMG episodes per hour [31]. The certainty of evidence was rated “moderate” in trials with repeated‐night assessments, while single‐night trials were graded “low” because of methodological limitations and variability in findings [31]. Florjanski et al. evaluated the certainty of evidence for the intervention biofeedback using the GRADE approach, with muscle activity as the outcome [29]. Among the included studies, three were rated “very low”, six “low”, and one “moderate” [29]. The downgrading of evidence was mainly related to limitations in study design, variability between studies, and indirectness of the evidence [29]. Amorim et al. evaluated the certainty of evidence for electrotherapeutic interventions, including biofeedback [33]. The certainty of evidence was rated “very low”, suggesting limited confidence in the effects of these interventions on muscle activity and pain in individuals with bruxism [33].

## 4 Discussion

Biofeedback therapy is a behavioral approach that aims to modify physiological processes through self‐regulation of muscle activity. In the management of TMD and bruxism, it is considered a non‐invasive alternative to conventional treatments. This umbrella review synthesized evidence on biofeedback‐based therapy for patients with myofascial orofacial pain, with or without bruxism, focusing on pain reduction as the primary outcome and muscle activity as a secondary outcome. The therapeutic principle of biofeedback is based on operant conditioning, in which various feedback methods allow patients to consciously modulate involuntary muscle tension [18, 19]. In line with contemporary TMD frameworks, this is clinically relevant because pain‐related TMD is increasingly conceptualized within a biopsychosocial model in which motor behavior (e.g., sustained clenching), psychosocial modulation, and central pain processing may interact; therefore, changes in muscle activation do not necessarily translate into proportional changes in perceived pain [4, 5, 6, 7, 15].

Chronic hyperactivity of the masticatory muscles has been associated with sustained nociceptive input and central sensitization, contributing to persistent orofacial pain [6, 7, 15, 43]. Biofeedback provides real‐time information on muscle activity and facilitates voluntary relaxation. Across the included systematic reviews, four reported reductions in masticatory muscle activity [29, 31, 33, 34], indicating that biofeedback can modulate neuromuscular output. Nevertheless, neuromuscular modulation and analgesia represent distinct clinical endpoints. Central sensitization and altered pain modulation may sustain symptoms despite measurable reductions in peripheral muscle activity, particularly in chronic pain phenotypes [6, 7, 15, 43].

Interpretation of these findings must be contextualized within the methodological limitations of the available evidence. The majority of included systematic reviews were rated as critically low confidence according to AMSTAR 2 [24]. Therefore, apparent consistency across reviews reflects repeated synthesis of a limited and methodologically weak pool of primary studies rather than independent confirmation of effect. Moreover, substantial variation existed across reviews in eligibility criteria, diagnostic definitions, intervention characterization, outcome measures, and synthesis strategies. Such heterogeneity increases the likelihood that similar conclusions arise from structurally different evidence bases [21, 24].

While the review identified various biofeedback modalities, including visual, auditory, and cranial electrotherapy stimulation (CES), the current evidence does not permit conclusions about their comparative effectiveness. This limitation restricts the ability to inform clinical decision‐making on the optimal modality. In addition, several reviews combined biofeedback modalities with conceptually distinct interventions (e.g., education, counselling, relaxation training, or CBT), limiting attribution of observed effects to biofeedback as an isolated component [27, 30, 32, 35, 36].

Evidence on pain reduction was heterogeneous. Results were inconclusive in seven studies [27, 29, 31, 33, 34, 35, 36, 37, 42], neutral in two [30, 34], and positive in one [28]. In three studies combining biofeedback with CBT, positive effects on pain were reported [27, 30, 32]. However, these improvements cannot be attributed solely to biofeedback. This pattern is consistent with the broader clinical logic that self‐management interventions targeting behavioral and cognitive contributors to persistent pain may be more likely to produce patient‐relevant analgesic benefit than interventions that primarily target muscle activation alone [4, 15, 27, 30].

Importantly, even where statistically significant reductions in pain were reported, clinical meaningfulness was not consistently evaluated across reviews. The extent to which observed mean differences met established minimal clinically important difference (MCID) thresholds remains unclear, limiting translation into clinical decision‐making. Notably, where patient‐important thresholds were explicitly addressed, clinically meaningful improvement was reported primarily for CBT augmented with biofeedback rather than for biofeedback alone; for example, the network meta‐analysis reported clinically meaningful improvement proportions for combined approaches, while evidence for biofeedback alone was low/very low certainty and lacked consistent responder reporting [27, 42].

Notably, in the review by Miron et al., the positive outcome for pain reduction was based on a single primary study with a small sample size, limiting the reliability of this finding [28]. Similarly, Iwasaki et al. included only one study using biofeedback, applied in combination with CBT, which also reported positive effects on pain [32]. Accordingly, these findings should be interpreted as signals of potential benefit under specific combined, behaviorally oriented care models rather than as confirmation of a standalone analgesic effect attributable to biofeedback [27, 30, 32].

Studies including patients with bruxism [28, 30, 31, 34] are challenging to interpret because bruxism is not inherently painful [14]. Therefore, any reported pain reduction may reflect improvements in TMD‐related pain secondary to muscle overactivity rather than a general effect applicable to myofascial pain. Evidence comparing biofeedback with other interventions for myofascial orofacial pain is limited. One systematic review [27] reported that CBT combined with biofeedback appeared more effective for pain reduction than CBT alone or other treatments such as jaw mobilization, occlusal splints, or medications. CBT alone was less effective, and it can be hypothesized that biofeedback enhances neuromuscular self‐regulation, thereby complementing the behavioral strategies of CBT. Because this conclusion is drawn from a single review, it should be interpreted with caution, and further high‐quality studies are needed to confirm these findings. Biofeedback reliably reduced masticatory muscle activity, indicating its potential for neuromuscular modulation. However, the inconsistent effects on pain suggest that additional factors, such as central sensitization, psychosocial influences, and comorbidities, likely contribute to pain perception and may not be fully addressed by biofeedback alone [4, 5, 6, 7, 15]. This aligns with contemporary pain models in which chronic TMD may overlap with centralized pain conditions; therefore, subgroup‐level treatment response may depend on baseline phenotype (e.g., pain chronicity, psychosocial burden, and signs of central sensitization), which was not consistently stratified in the included primary trials or reviews [1, 2, 7, 15]. Decreases in muscle activity may help reduce functional limitations or prevent bruxism‐related tissue strain, but the extent to which these changes lead to significant pain relief remains uncertain.

Among the eleven SRs analysed, only three conducted a meta‐analysis [30, 31, 34] and one a network meta‐analysis [27] to synthesize findings. However, in two of these [31, 34], the analyses did not address the outcomes relevant to this review and instead focused on SB episodes. Interpretation of findings is limited by heterogeneity across studies, which led most reviews to rely on descriptive rather than quantitative synthesis [28, 29, 32, 33, 35, 36, 37]. Pain was assessed using a variety of instruments. Differences in baseline pain duration among patients hindered direct comparisons. The application of biofeedback varied in modality, intensity, frequency, and whether it was delivered alone or combined with other interventions. This heterogeneity, together with limited reporting and the lack of method‐specific outcome data, made it impossible to determine both the independent effects of biofeedback and the specific advantages or disadvantages of the different modalities. From an evidence‐interpretation standpoint, this heterogeneity is clinically consequential because it mixes trials targeting potentially different mechanisms (sleep bruxism episode suppression, daytime clenching awareness, autonomic arousal reduction) under a single “biofeedback” label, thereby diluting mechanism‐specific inferences [14, 27, 29, 31, 34].

Reporting on study registration and protocol details was rare; only three reviews [27, 30, 31] reported protocol registration, raising concerns about transparency and risk of bias. The reliability of the synthesized evidence must be interpreted cautiously given methodological weaknesses across reviews. AMSTAR‐2 [24] ratings indicated predominantly “critically low” methodological quality, with only two reviews rated as “low”. The moderate overlap among primary studies (CCA = 8.48%) shows some reviews used the same research. While this does not imply high redundancy, it might overstate confidence if viewed as separate confirmations. Therefore, consistency across reviews indicates multiple assessments of a limited set of trials, not stronger proof. This concern is well‐recognized in umbrella review methodology: overlapping primary studies can create an “illusion of replication,” particularly when conclusions are reiterated across reviews that draw on the same small trial base [21, 22]. Certainty ratings reported in this overview reflect the assessments performed by the authors of the included systematic reviews and were not re‐evaluated at the umbrella review level. Consequently, variations in GRADE application and interpretation across reviews may have influenced the overall certainty profile presented here.

From a clinical perspective, biofeedback appears promising for reducing masticatory muscle hyperactivity and may serve as an adjunctive therapy for patients with myofascial pain or bruxism related to excessive muscle activity. Nevertheless, caution should be exercised regarding this observed consistency in decreased muscle activity due to limitations in the review and overlapping studies. Given the limited and heterogeneous evidence, biofeedback should be considered an adjunctive rather than a standalone approach for patients with chronic TMD‐related pain [4, 15, 27, 30]. Future studies should aim to establish standardized biofeedback protocols, employ consistent EMG and pain assessment methods, and include long‐term follow‐up to evaluate the durability of therapeutic effects. To improve clinical interpretability, future RCTs should predefine patient‐centered responder outcomes (including MCID‐based pain response), report adverse events and acceptability, and stratify or adjust analyses by chronicity and psychosocial risk profiles (e.g., catastrophizing or comorbid pain conditions), consistent with biopsychosocial TMD models [1, 2, 4, 15, 27, 42]. Given the moderate overlap observed in this overview, future efforts should prioritize high‐quality primary studies (RCTs) rather than additional SRs to generate more robust and reliable evidence [21, 22, 23, 24]. Future trials should focus on standardized EMG‐based biofeedback protocols and explore synergistic effects with behavioral therapies using rigorous methodological designs [27, 30].

## 5 Conclusions

From this umbrella review, the following conclusions can be drawn:

- The included systematic reviews were predominantly rated as critically low or low in methodological quality. The overall certainty of evidence regarding the efficacy of biofeedback for myofascial orofacial pain and bruxism is therefore limited. Consistency across reviews reflects synthesis of a small and partially overlapping set of primary studies rather than independent confirmation of effect.
- Biofeedback appears to reduce masticatory muscle activity. However, its effect on pain remains inconsistent. Variability in intervention protocols, outcome definitions, and follow up periods prevents firm conclusions regarding clinical effectiveness or durability. Evidence supporting reduction in muscle activity derives from limited and overlapping trials and must be interpreted with caution.
- Based on the current evidence, biofeedback cannot be recommended as an independent therapy for chronic TMD related pain. Its role is best considered adjunctive within a comprehensive biopsychosocial management approach. Future research should include adequately powered randomized controlled trials with standardized protocols, clearly defined patient‐centered outcomes, and long‐term follow up to determine therapeutic efficacy.

## Author Contributions

**Celine Beriger:** conceptualization, validation, visualization, data curation, writing – original draft, writing – review and editing, investigation. **Kathrin Kohout:** conceptualization, validation, formal analysis, investigation, visualization, data curation. **Aleksandra Zumbrunn:** conceptualization, validation, data curation. **Edwin Ruales‐Carrera:** validation, formal analysis, writing – original draft, writing – review and editing, visualization, investigation. **Tan Fırat Eyüboğlu:** validation, writing – review and editing, visualization. **Mutlu Özcan:** conceptualization, methodology, validation, formal analysis, supervision, project administration, writing – original draft, writing – review and editing, data curation, investigation, visualization.

## Funding

The authors have nothing to report.

## Ethics Statement

The authors have nothing to report.

## Consent

The authors have nothing to report.

## Conflicts of Interest

The authors declare no conflicts of interest.

## Supporting information


**Appendix S1:** Search strategy used for each of the databases.
**Appendix S2:** Supplementary Tables S1–S5.
**Table S1:** Excluded articles with reason (*n* = 45).
**Table S2:** PICOS analysis (*n* = 11).
**Table S3:** Bibliometric characteristics (*n* = 11).
**Table S4:** Matrix of evidence with included studies per included systematic review.
**Table S5:** Biofeedback characteristics of included studies.

## Data Availability

Datasets used and/or analysed during this study are available from the authors upon reasonable request.
