# Supplementary material for: Biofeedback‐Based Therapy in Patients With Myofascial Orofacial Pain and Bruxism: An Umbrella Review
Source: J Oral Rehabil. 2026 Sep 9;53(12):2439–55. doi: 10.1111/joor.70303 (PMC13755666; doi:10.1111/joor.70303)
Supplement: Supplementary file 1 [file JOOR-53-2439-s001.docx]

**Supplementary Appendices**

**Supplementary Appendix 1: Search strategy used for each of the Databases.**

| Database | Search |
| --- | --- |
| EMBASE | #1 AND #2 AND ([english]/lim OR [german]/lim) NOT (([infant]/lim OR [child]/lim OR [adolescent]/lim) NOT ([adult]/lim OR [aged]/lim)) NOT [conference abstract]/lim  #1 AND #2 AND ([english]/lim OR [german]/lim) NOT (([infant]/lim OR [child]/lim OR [adolescent]/lim) NOT ([adult]/lim OR [aged]/lim)) AND [conference abstract]/lim  #1 AND #2 AND ([english]/lim OR [german]/lim) NOT (([infant]/lim OR [child]/lim OR [adolescent]/lim) NOT  ([adult]/lim OR [aged]/lim))  #1 AND #2 AND ([english]/lim OR [german]/lim)  #1 AND #2  'biofeedback'/de OR 'autoregulation'/de OR 'feedback system'/de OR 'sensory feedback'/exp OR 'bio feed back':ti,ab OR 'bio-feedback':ti,ab OR 'biofeed-back':ti,ab OR biofeedback:ti,ab OR 'myobiofeedback':ti,ab OR 'myofeedback':ti,ab OR 'auto regulation':ti,ab OR autoregulation:ti,ab OR 'self regulation':ti,ab OR 'self care':ti,ab OR 'self management':ti,ab OR 'self treatment':ti,ab OR selfregulation:ti,ab OR selfcare:ti,ab OR selfmanagement:ti,ab OR selftreatment:ti,ab OR ((feedback NEAR/5 (train* OR mechanism* OR factor* OR system* OR correct* OR masseter OR mastic*)):ti,ab)  'bruxism'/exp OR 'temporomandibular joint disorder'/exp OR 'costen syndrome':ti,ab OR (((craniomandibular OR temporomandibular OR 'temporo mandibular' OR 'cranio mandibular') NEAR/3 (disorders OR dysfunction* OR disease OR syndrome*)):ti,ab) OR bruxi*:ti,ab OR bruxo*:ti,ab OR bruxer*:ti,ab OR ((clench* NEAR/3 (habit* OR teeth OR tooth)):ti,ab) OR (((dental OR tooth OR teeth) NEAR/3 grind*):ti,ab) OR (((orofacial OR myofacial) NEAR/3 (pain OR syndrome*)):ti,ab) OR (('myofascial pain'/exp OR 'face pain'/exp OR (((myofascial OR facial) NEAR/3 (pain OR syndrome* OR neuralgia*)):ti,ab) OR myalgia:ti,ab OR facialgia:ti,ab) AND ('jaw disease'/exp OR 'mouth pain'/exp OR 'masticatory muscle'/exp OR jaw:ti,ab OR mastic*:ti,ab OR masseter:ti,ab OR mouth:ti,ab OR temporomandibul*:ti,ab OR craniomandibul*:ti,ab OR 'temporo mandibul*':ti,ab OR 'cranio mandibul*':ti,ab)) |
| MEDLINE | exp Bruxism/ or exp craniomandibular disorders/ or exp *temporomandibular joint disorders/ or "Costen syndrome".ti,ab. or ((craniomandibular or temporomandibular or temporo-mandibular or cranio-mandibular) adj3 (disorder* or dysfunction* or disease* or syndrome*)).ti,ab. or (bruxi* or bruxo* or bruxer*).ti,ab. or (clench* adj3 (habit* or teeth or tooth)).ti,ab. or ((dental or tooth or teeth) adj3 grind*).ti,ab. or ((orofacial or myofacial) adj3 (pain or syndrome*)).ti,ab. or ((exp *Myofascial Pain Syndromes/ or Facial Pain/ or ((myofascial or facial) adj3 (pain or syndrome* or neuralgia*)).ti,ab.) and (exp Jaw Diseases/ or masticatory muscles/ or exp masseter muscle/ or (jaw or mastic* or masseter or mouth or temporomandibul* or craniomandibul* or temporo-mandibul* or cranio-mandibul*).ti,ab.))  Biofeedback, Psychology/ or Feedback, Sensory/ or ("bio feed back" or "bio-feedback" or "biofeed-back" or biofeedback or "myobiofeedback" or "myofeedback").ti,ab. or (auto-regulation or autoregulation or "self regulation" or self-care or self-management or self-treatment or selfregulation or selfcare or selfmanagement or selftreatment).ti,ab. or (feedback adj5 (train* or mechanism* or factor* or system* or correct* or masseter or mastic*)).ti,ab.  1 and 2  limit 3 to english language  ("30620145" or "38101924" or "36674315" or "31099036" or "3607094" or "16570467" or "23446073").ui.  4 and 5 |
| Cochrane | #1 AND #2  ("bio feed back" OR "bio-feedback" OR "biofeed-back" OR biofeedback OR "myobiofeedback" OR "myofeedback"):ti,ab,kw OR (auto-regulation OR autoregulation OR "self regulation" OR self-care OR self-management OR self-treatment OR selfregulation OR selfcare OR selfmanagement OR selftreatment):ti,ab,kw OR (feedback NEAR/5 (train* OR mechanism* OR factor* OR system* OR correct* OR masseter OR mastic*)):ti,ab,kw  "Costen syndrome":ti,ab,kw OR ((craniomandibular OR temporomandibular OR temporo-mandibular OR cranio-mandibular) NEAR/3 (disorder* OR dysfunction* OR disease* OR syndrome*)):ti,ab,kw OR (bruxi* OR bruxo* OR bruxer*):ti,ab,kw OR (clench* NEAR/3 (habit* OR teeth OR tooth)):ti,ab,kw OR ((dental OR tooth OR teeth) NEAR/3 grind*):ti,ab,kw OR ((orofacial OR myofacial) NEAR/3 (pain OR syndrome*)):ti,ab,kw  OR  (( ((myofascial OR facial) NEAR/3 (pain OR syndrome* OR neuralgia*)):ti,ab,kw OR (myalgia OR facialgia):ti,ab,kw) AND (jaw OR mastic* OR masseter OR mouth OR temporomandibul* OR craniomandibul* OR temporo-mandibul* OR cranio-mandibul*):ti,ab,kw) |
| Web of Science | TS="Costen syndrome" OR TS=((craniomandibular OR temporomandibular OR temporo-mandibular OR cranio-mandibular) NEAR/3 (disorder* OR dysfunction* OR disease* OR syndrome*)) OR TS=(bruxi* OR bruxo* OR bruxer*) OR TS=(clench* NEAR/3 (habit* OR teeth OR tooth)) OR TS=((dental OR tooth OR teeth) NEAR/3 grind*) OR TS=((orofacial OR myofacial) NEAR/3 (pain OR syndrome*))  OR  (( TS=((myofascial OR facial) NEAR/3 (pain OR syndrome* OR neuralgia*)) OR TS=(myalgia OR facialgia)) AND TS=(jaw OR mastic* OR masseter OR mouth OR temporomandibul* OR craniomandibul* OR temporo-mandibul* OR cranio-mandibul*))  TS=("bio feed back" OR "bio-feedback" OR "biofeed-back" OR biofeedback OR "myobiofeedback" OR "myofeedback") OR TS=(auto-regulation OR autoregulation OR "self regulation" OR self-care OR self-management OR self-treatment OR selfregulation OR selfcare OR selfmanagement OR selftreatment) OR TS=(feedback NEAR/5 (train* OR mechanism* OR factor* OR system* OR correct* OR masseter OR mastic*))  #2 AND #1  #2 AND #1 and English or German (Languages) |
| LILACS | (("Costen syndrome" OR ((craniomandibular OR temporomandibular OR temporo-mandibular OR cranio-mandibular) AND (disorder* OR dysfunction* OR disease* OR syndrome*)) OR (bruxi* OR bruxo* OR bruxer*) OR (clench* AND (habit* OR teeth OR tooth)) OR ((dental OR tooth OR teeth) AND grind*) OR ((orofacial OR myofacial) AND (pain OR syndrome*)) OR ((myalgia OR facialgia) AND (jaw OR mastic* OR masseter OR mouth OR temporomandibul* OR craniomandibul* OR temporo-mandibul* OR cranio-mandibul*)))) AND ("bio feed back" OR "bio-feedback" OR "biofeed-back" OR biofeedback OR "myobiofeedback" OR "myofeedback" OR auto-regulation OR autoregulation OR "self regulation" OR self-care OR self-management OR self-treatment OR selfregulation OR selfcare OR selfmanagement OR selftreatment OR (feedback AND (train* OR mechanism* OR factor* OR system* OR correct* OR masseter OR mastic*))) AND db:("LILACS") AND la:("en") AND instance:"lilacsplus" AND instance:"lilacsplus"  ((("Costen syndrome" OR ((craniomandibular OR temporomandibular OR temporo-mandibular OR cranio-mandibular) AND (disorder* OR dysfunction* OR disease* OR syndrome*)) OR (bruxi* OR bruxo* OR bruxer*) OR (clench* AND (habit* OR teeth OR tooth)) OR ((dental OR tooth OR teeth) AND grind*) OR ((orofacial OR myofacial) AND (pain OR syndrome*)) OR ((myalgia OR facialgia) AND (jaw OR mastic* OR masseter OR mouth OR temporomandibul* OR craniomandibul* OR temporo-mandibul* OR cranio-mandibul*)))) AND ("bio feed back" OR "bio-feedback" OR "biofeed-back" OR biofeedback OR "myobiofeedback" OR "myofeedback" OR auto-regulation OR autoregulation OR "self regulation" OR self-care OR self-management OR self-treatment OR selfregulation OR selfcare OR selfmanagement OR selftreatment OR (feedback AND (train* OR mechanism* OR factor* OR system* OR correct* OR masseter OR mastic*))) ) AND db:("LILACS") AND instance:"lilacsplus"  (("Costen syndrome" OR ((craniomandibular OR temporomandibular OR temporo-mandibular OR cranio-mandibular) AND (disorder* OR dysfunction* OR disease* OR syndrome*)) OR (bruxi* OR bruxo* OR bruxer*) OR (clench* AND (habit* OR teeth OR tooth)) OR ((dental OR tooth OR teeth) AND grind*) OR ((orofacial OR myofacial) AND (pain OR syndrome*)) OR ((myalgia OR facialgia) AND (jaw OR mastic* OR masseter OR mouth OR temporomandibul* OR craniomandibul* OR temporo-mandibul* OR cranio-mandibul*)))) AND ("bio feed back" OR "bio-feedback" OR "biofeed-back" OR biofeedback OR "myobiofeedback" OR "myofeedback" OR auto-regulation OR autoregulation OR "self regulation" OR self-care OR self-management OR self-treatment OR selfregulation OR selfcare OR selfmanagement OR selftreatment OR (feedback AND (train* OR mechanism* OR factor* OR system* OR correct* OR masseter OR mastic*))) |
| SCOPUS | #1 AND #2 AND ([english]/lim OR [german]/lim)  #1 AND #2  TITLE-ABS("bio feed back" OR "bio-feedback" OR "biofeed-back" OR biofeedback OR "myobiofeedback" OR "myofeedback") OR TITLE-ABS(auto-regulation OR autoregulation OR "self regulation" OR self-care OR self-management OR self-treatment OR selfregulation OR selfcare OR selfmanagement OR selftreatment) OR TITLE-ABS(feedback W/5 (train* OR mechanism* OR factor* OR system* OR correct* OR masseter OR mastic*))  TITLE-ABS("Costen syndrome") OR TITLE-ABS((craniomandibular OR temporomandibular OR temporo-mandibular OR cranio-mandibular) W/3 (disorder* OR dysfunction* OR disease* OR syndrome*)) OR TITLE-ABS(bruxi* OR bruxo* OR bruxer*) OR TITLE-ABS(clench* W/3 (habit* OR teeth OR tooth)) OR TITLE-ABS((dental OR tooth OR teeth) W/3 grind*) OR TITLE-ABS((orofacial OR myofacial) W/3 (pain OR syndrome*))  OR (( TITLE-ABS((myofascial OR facial) W/3 (pain OR syndrome* OR neuralgia*)) OR TITLE-ABS(myalgia OR facialgia)) AND TITLE-ABS(jaw OR mastic* OR masseter OR mouth OR temporomandibul* OR craniomandibul* OR temporo-mandibul* OR cranio-mandibul*)) |
| Clinicaltrial.gov | craniomandibular OR temporomandibular OR temporo-mandibular OR cranio-mandibular  orofacial OR myofascial  Grinding teeth  Bruxism  biofeedback OR bio-feedback  biofeedback OR bio-feedback |
| ICTRP | bruxism OR craniomandibular OR temporomandibular OR temporo-mandibular OR cranio-mandibular OR orofacial OR myofascial  biofeedback OR bio-feedback |

**Supplementary Appendix 2: Supplementary Tables**

**Table S1.** Excluded articles with reason (n=45).

| Study | Reason for exclusion |
| --- | --- |
| Zhu et al. (2024) | Review did not meet the PICO criteria |
| Ainoosah et al. (2024) | Review did not meet the PICO criteria |
| Soares-Silva et al. (2024) | Another type of study than systematic reviews (SRs) |
| Rampazo et al. (2024) | Review did not meet the PICO criteria |
| Mancini et al. (2024) | Review did not meet the PICO criteria |
| Arribas-Pascual et al. (2023) | Another type of study than systematic reviews (SRs) |
| Vieira et al. (2023) | Review did not meet the PICO criteria. Excluded due to lack of primary outcome (pain) as defined by our PICO framework. |
| Ceron et al. (2023) | Another type of study than systematic reviews (SRs) |
| Palmer et al. (2023) | Review did not meet the PICO criteria |
| Batista et al. (2022) | Review did not meet the PICO criteria |
| Minakuchi et al. (2022) | Review did not meet the PICO criteria. Excluded due to lack of our primary outcome (pain) as defined by our PICO framework. |
| Goldstein et al. (2021) | Another type of study than systematic reviews (SRs) |
| Oliveira et al. (2020) | Another type of study than systematic reviews (SRs) |
| Bussadori et al. (2020) | Another type of study than systematic reviews (SRs) |
| De Melo et al. (2020) | Review did not meet the PICO criteria |
| Fouda (2020) | Use of biofeedback not clearly reported or isolated. |
| Ferrando Garcia (2019) | Another type of study than systematic reviews (SRs) |
| Kondo et al. (2019) | Another type of study than systematic reviews (SRs) |
| Patel et al. (2019) | Review did not meet the PICO criteria |
| Yadav et al. (2018) | Another type of study than systematic reviews (SRs) |
| Pimentel et al. (2018) | Another type of study than systematic reviews (SRs) |
| Mesko et al. (2017) | Another type of study than systematic reviews (SRs) |
| Story et al. (2016) | Use of biofeedback not clearly reported or isolated. |
| Al-Ani et al. (2016) | Withdrawn |
| Randhawa et al. (2016) | No full text available |
| Stechman-Neto et al. (2016) | Review did not meet the PICO criteria |
| Wieckiewicz et al. (2015) | Another type of study than systematic reviews (SRs) |
| Manfredini et al. (2015) | Another type of study than systematic reviews (SRs) |
| Patel et al. (2014) | Another type of study than systematic reviews (SRs) |
| Kotiranta et al. (2014) | Another type of study than systematic reviews (SRs) |
| Ilovar et al. (2014) | Another type of study than systematic reviews (SRs). Protocol no study available. |
| De Freitas et al. (2013) | Another type of study than systematic reviews (SRs) |
| Januzzi et al. (2013) | Review did not meet the PICO criteria |
| Aggarwal et al. (2011) | Withdrawn |
| List & Axelsson (2010) | Another type of study than systematic reviews (SRs) |
| Aggarwal et al. (2010) | Review did not meet the PICO criteria |
| Turp et al. (2007) | Another type of study than systematic reviews (SRs) |
| Rinchuse & McMinn (2006) | Another type of study than systematic reviews (SRs) |
| Al-Ani et al. (2005) | Review did not meet the PICO criteria |
| Al-Ani et al. (2004) | Withdrawn |
| Ernst (2003) | Another type of study than systematic reviews (SRs) |
| Tsukiyama et al. (2001) | Another type of study than systematic reviews (SRs) |
| Crider & Glaros (1999) | Another type of study than systematic reviews (SRs) |
| Carroll & Seers (1998) | Review did not meet the PICO criteria |
| Mohl et al. (1990) | Another type of study than systematic reviews (SRs) |

**Table S2.** PICOS analysis (n=11).

| Author (year) | Population (N) | Diagnosis method | Interventions and comparators | Outcome(s) | Follow up range | Primary studies |
| --- | --- | --- | --- | --- | --- | --- |
| Yao et al. (2023) | Adults (>18 years) with chronic pain associated with TMD | RDC/TMD, Clinical diagnosis | Intervention: any intervention currently in use for chronic pain associated with TMD (23 conservative, 15 pharmacological, seven combination of pharmacological and conservative and 13 surgical)  Comparison: placebo/sham | Pain, physical functioning, emotional functioning, role functioning, social functioning, sleep quality, and adverse events | In studies with multiple follow-up duration, data were extracted from the longest available follow-up (median follow-up was 12 weeks) | Qualitative synthesis:  RCT (233)  (Biofeedback (2 RCT) CBT combined with biofeedback (2 RCT))  Quantitative synthesis:  RCT (153) |
| Miron et al. (2022) | Adults (>18 years) with nocturnal bruxism | Clinical diagnosis or standard clinical diagnosis for nocturnal bruxism (questionnaires, clinical evaluations, EMG, PSG) | Intervention: different treatment interventions for nocturnal bruxism  Comparison: comparison between the different treatment interventions in use for nocturnal bruxism (Bergmann et al. (2020) uses biofeedback as intervention and conventional occlusal splint (AOS) as the comparison) | Pain, quality of life, episodes of nocturnal bruxism | Three months treatment  Follow-up not reported | Qualitative synthesis:  RCT (9)  (Biofeedback (1 RCT)) |
| Florjanski et al. (2019) | Patients of any age with clinical diagnosis of TMD treated with biofeedback   - Age of patients ranged from 20 years to 60 years! | AASM, RDC/TMD, Self-report, No data, PSG, EMG | Intervention: Two biofeedback groups (biofeedback, CES)  Comparison: TENS, control group, occlusal splint | Muscle activity, pain, bruxism episodes | Only two out of 10 studies have a follow-up (Conti et al. (2014): five nights, Raphael et al. (2013): two weeks) | (10)  RCT  Crossover Studies  Single-Blinded |
| Iwasaki et al. (2018) | Adults (>18 years), untreated and diagnosed according to RDC/TMD | RDC/TMD Axis I or Axis II based on clinical examination | Intervention: CBT, CBT combined with biofeedback, CBT plus standard treatment  Comparison: stress management, nonintervention, standard treatment alone | Pain (VAS, other methods) | Different follow-ups in each study (12 months for CBT augmented with biofeedback) | Qualitative synthesis:  RCT (6)  (CBT combined with biofeedback (1 RCT)) |
| Amorim et al. (2018) | Patients with SB or AB  But the average mean values and respective standard deviations for studies relevant to biofeedback, all involved adults.  Unweighted mean ± SD ≈ 32.2 ± 5.5 | Clinical assessment, EMG, PSG | Intervention: Physical therapy, including electrotherapy (mainly biofeedback, but also TENS and MENS), CBT, therapeutic exercises, acupuncture, postural awareness, muscular relaxation, and massage  Comparison: control conditions | Muscle pain, muscle activity, mouth opening, anxiety, stress, depression, sleep and oral health quality, TMD, head posture | Missing information from primary studies | Qualitative synthesis:  RCT (14)  CCT (2)  CT (8)  (Biofeedback (11 RCT), CBT combined with biofeedback (1 RCT)) |
| Medlicott & Harris (2006) | Patients with acute (< 6 months) or chronic (> 6 months) TMD | RMC/TMD Axis I | Intervention: various physical therapies  Comparison: sham, control, waiting list control, TENS, other physical therapies | Pain, ROM, disability and function, joint noise, tenderness, psychological factors | Different follow-ups in each study (none to four years) | Qualitative synthesis:  RCT (22)  Pretest-Prosttest (4)  Case Serie (2)  Randomized, placebo-controlled, crossover trial  (1)  One group with randomized order of treatments (1)  (Biofeedback (7 RCT)) |
| McNeely et al. (2006) | Adults (>18 years) with TMD, musculoskeletal dysfunction, pain impairment, no previous surgery in temporomandibular region, no comorbid conditions | RDC/TMD, own diagnostic criteria based on symptoms | Intervention: physical therapy (exercise, acupuncture, electrophysical modalities, manual therapy, mechanical therapy device)  Comparison: placebo, controlled comparison intervention, standard care (comparison for biofeedback: relaxation therapy, occlusal splint therapy, TENS) | Primary: pain, ROM, oral function  Secondary: EMG activity, patient satisfaction | Not reported  Funch and Gale (1984): post-treatment and 2 years | RCT (30)  (Biofeedback (4 RCT)) |
| Jedel & Carlsson (2003) | Patients with signs or symptoms of TMD | RDC/TMD, IHS | Intervention: «Studies evaluating a modality as adjuncts to a management program if a control received the same management program» (1. splint therapy, 2. biofeedback, 3. biofeedback as an adjunct)  Comparison: 1. biofeedback, 2. experimental control, 3. three control groups  Comparison only listed for biofeedback relevant interventions/comparisons | Pain, subjective improvement of symptoms and/or specific functional status | Not reported | CCT (7)  (Biofeedback (2 CCT) CBT combined with biofeedback (1CCT)) |
| Aggarwal et al. (2019) | Adults (>18y) with chronic orofacial pain (TMD, atypical fascial pain, atypical odontalgia, burning mouth syndrome)  N (all 14 RCTs included) = 779  N (six studies with biofeedback) = 415 | Various across primary studies | Intervention: self-management (patient manual, between session work, physical self-regulation (jaw posture relaxation and habit reversal, biofeedback, breathing techniques), psychological (cognitive therapy, behavior therapy))  Comparison: usual care (conservative treatment including patient education, therapeutic counselling and flat-plane intraoral appliance) | Pain intensity: short and long term (VAS, validated categorial scale); muscle palpation pain: long term; activity interference and pain impact on activities: long term (pain inventory and multidimensional pain inventory); depression: long term (validated scales) | Not reported | RCT (14)  nRCT (0)  (Biofeedback and CBT combined with biofeedback (6 RCT)) |
| Jokubauskas & Baltrušaitytė (2018) | Children & Adults with SB  (N=86)  Age of patients >18 years | Various across primary studies in terms of SB severity rate and demographic qualities (questionnaires and/or clinical assessment, night-time EMG monitoring, PSG evaluation) | Intervention: Biofeedback (CES, audio feedback, maxillary biofeedback splint)  Comparison: other treatment | Change in frequency of SB episode, bursts and motor activities; change in sleep and life quality, stress levels, discomfort, orofacial-pain features and patience compliance | Various across primary studies | Qualitative synthesis:  RCT (4)  nRCT (2)  Quantitative synthesis:  RCT (1 of 4) |
| Wang et al. (2013) | Patients of any age with SB (N=240)  Age of patients >18 years | EMG record, except one study used the abrasion degree of a bite plate-like device | Biofeedback (CES, audio feedback, nocturnal audio feedback, diurnal feedback, visual feedback); CBT with partial biofeedback | Frequency of SB events (EMG); bruxism-related muscle EMG activity; pain level (number of painful muscles, characteristic pain intensity, McGill pain questionnaire); sleep quality improvement | Not explicitly reported | RCT (6)  nRCT (0)  CCT (1) |

TMD (Temporomandibular Dysfunction); VAS (Visual Analog Scale); RCT (Randomized Controlled Trial); nRCT (Non- Randomized Controlled Trial); CCT (Controlled Clinical Trial); SB (Sleep Bruxism); EMG (electromyography); PSG (polysomnography); CES (Cranial Electrotherapy Stimulation); CBT (Cognitive Behavioral Therapy); ROM (Range of motion); TENS (Transcutaneous Electrical Nerve Stimulation)

| **Table S3.** Bibliometric characteristics (n=11). | | | | | | | | |
| --- | --- | --- | --- | --- | --- | --- | --- | --- |
| Author (year)  Journal impact factor | Country of the authors | 1. Cochrane review 2. GRADE 3. Meta-analysis 4. Protocol registration | Database(s) searched  Date assessed as up to date | 1. Grey literature 2. Experts | Risk of bias assessment tool | Software used in the MA | Number of studies included in the MA | Conflict of interest |
| Yao et al. (2023)  93.7 | Canada, China, USA, Colombia, Singapore, Switzerland, Norway | 1. Yes 2. Yes 3. Yes 4. Yes (PROSPERO) | MEDLINE, EMBASE, CINAHL, CENTRAL, and SCOPUS  Up to May 2021, again in January 2023 | 1. No 2. Yes | The cochrane risk of bias tool (RoB1) | STATA (MP Edition) | 153 | No |
| Miron et al. (2022)  - | Romania | 1. Yes 2. No 3. No 4. No | PubMed, ScienceDirect, Google Scholar  2012 up to 2022 | 1. No 2. No | The cochrane risk of bias tool | - | - | Not reported |
| Florjanski et al. (2019)  2.9 | Poland | 1. No 2. Yes 3. No 4. No | MEDLINE, Scopus, Web of Science, CINAHL  Between January 1998 and January 2019) | 1. No 2. No | - | - | - | No |
| Iwasaki et al. (2018)  0.300 | Japan | 1. No 2. No 3. No 4. No | Pub med  Up to March 10, 2016 | 1. No 2. No | The cochrane risk of bias tool | - | 2 (only CBT no biofeedback) | No |
| Amorim et al. (2018)  1.4 | Brazil | 1. No 2. Yes 3. No 4. Not reported | Medline, Excerpta Medica Database, Cumulative Index of Nursing and Allied Health Literature, Physiotherapy Evidence Database, SPORTDiscus, Scientific Electronic Library Online, Web of Science, Cochrane Library, Scopus, and Literatura Latino-Americana e do Caribe em Ciências da Saúde  Not reported (included studies published between January 1990 and September 2015) | 1. Yes 2. No | PEDro Scale, Jadad Scale | - | - | No |
| Medlicott & Harris (2006)  3.3 |  | 1. No 2. No 3. No 5. No | MEDLINE, CINAHL, CENTRAL  Between 1966 and January 2005 | 1. No 3. No |  | - | - | Not reported |
| McNeely et al. (2006)  3.3 | Canada | 1. No 2. No 3. No 6. No | MEDLINE (1966 to February 2005), EMBASE (1988 to February 2005), Cochrane Library and Best Evidence (1991 to 2005), ISI Web of Science (1965 to March 3, 2005), PubMed (1966 to March 3, 2005), Lilacs (1982 to March 3, 2005), EBM reviews Cochrane Central Register of Controlled Trials (1991 to 2005), and CINAHL (1982 to February, 2005) | 1. Yes 4. Yes | Jadad Scale | - | - | Not reported |
| Jedel & Carlsson (2003)  1.2 | Sweden | 1. No 2. No 3. No 7. No | Medline, CINAHL, EMBASE, PsycINFO and Cochrane Controlled Trials register  Up to may 2002 | 1. No 5. No | van Tulder criteria | - | - | Not reported |
| Aggarwal et al. (2019)  3.5 | UK | 1. No 2. Yes 3. Yes 8. Yes (PROSPERO) | The Cochrane Oral Health Group Trials Register, the Cochrane Central Register of Controlled Trials (CENTRAL), MEDLINE via OVID, EMBASE via OVID, PsycINFO via OVID, WHO International Clinical Trials Registry Platform, and Clinical Trials.gov  September 2017 | 1. Yes 2. No 6. | The cochrane risk of bias tool (RoB1) | Not reported | 14 | No |
| Jokubauskas & Baltrušaitytė (2018)  4.0 | Lithuania | 1. No 2. Yes 3. Yes 9. Yes | MEDLINE (searched via PubMed), EMBASE (searched via ScienceDirect), The Cochrane Library (Cochrane Central Register of Controlled Trials) and LILACS  After October 2012 up to 20 January 2018 | 1. Yes 7. No | GRADE criteria | Review Manager 5.3 (revman) | 3  (two additional studies from Wang et al. were included) | No |
| Wang et al. (2014)  2.1 | China | 1. No 2. No 3. Yes 10. No | Cochrane Central Register of Controlled Trials (CENTRAL), MEDLINE, Embase, ISI Web of Science, Chinese Biomedical Literature Database, and PsycINFO  October 2012 | 1. Yes 2. No 8. | The risk of bias assessment was performed based on the cochrane handbook for systematic reviews of interventions 5.1.0 | Review Manager 5.1 (revman) | 2 | No |

*GRADE (Grading of Recommendations Assessment Development and Evaluation); MA (Meta-analysis); PROSPERO (International Prospective Register of Systematic Reviews); RoB (Risk of Bias); ROBINS-I (Risk Of Bias In Non-Randomized Studies of Interventions); LILACS (Latin American and Caribbean Health Sciences Literature)*

**Table S4.** Matrix of evidence with included studies per included systematic review.

| Primary Studies | Systematic Reviews | | | | | | | | | | | |
| --- | --- | --- | --- | --- | --- | --- | --- | --- | --- | --- | --- | --- |
| *Study ID* | *Aggarwal et al. (2019)* | *Jokubauskas & Baltrušaitytė (2018)* | *Wang et al. (2013)* | *Yao et al. (2023)* | *Iwasaki et al. (2018)* | *Florjanski et al. (2019)* | *Medlicott & Harris (2006)* | *McNeely et al. (2006)* | *Amorim et al. (2018)* | *Miron et al. (2022)* | *Jedel & Carlsson (2003)* |  |
| Crockett et al. (1986) | *1* |  |  |  |  |  | *1* |  |  |  |  |  |
| Gardea et al. (2001) | *1* |  |  |  |  |  |  |  |  |  |  |  |
| Gatchel et al. (2006) | *1* |  |  |  | *1* |  |  |  |  |  |  |  |
| Shedden‐Mora et al. (2013) | *1* |  |  | *1* |  |  |  |  |  |  |  |  |
| Turk et al. (1993) | *1* |  |  | *1* |  |  | *1* |  |  |  |  |  |
| Turk et al. (1996) | *1* |  |  |  |  |  |  |  |  |  |  |  |
| Gu et al (2015) |  | *1* |  |  |  | *1* |  |  | *1* |  |  |  |
| Conti et al (2014) |  | *1* |  |  |  | *1* |  |  | *1* |  |  |  |
| Sato et al (2014) |  | *1* |  |  |  | *1* |  |  | *1* |  |  |  |
| *Jadidi et al (2013)* |  | *1* |  |  |  |  |  |  | *1* |  |  |  |
| Sumiya et al (2014) |  | *1* |  |  |  | *1* |  |  | *1* |  |  |  |
| Raphael et al (2013) |  | *1* |  |  |  | *1* |  |  | *1* |  |  |  |
| Kardachi (1978) |  |  | *1* |  |  |  |  |  |  |  |  |  |
| Casas (1982) |  |  | *1* |  |  |  |  |  |  |  |  |  |
| Pierce (1988) |  |  | *1* |  |  |  |  |  |  |  |  |  |
| Wieselmann-Penkner (2001) |  |  | *1* |  |  | *1* |  | *1* | *1* |  |  |  |
| Ommerborn (2007) |  |  | *1* |  |  |  |  |  | *1* |  |  |  |
| Jadidi (2008) |  |  | *1* |  |  | *1* |  |  | *1* |  |  |  |
| *Jadidi (2011)* |  |  | *1* |  |  |  |  |  | *1* |  |  |  |
| *Mishra (2000)* |  |  |  | *1* |  |  |  |  |  |  | *1* |  |
| *Barbosa (2019)* |  |  |  | *1* |  |  |  |  |  |  |  |  |
| *Criado et al. (2016)* |  |  |  |  |  | *1* |  |  |  |  |  |  |
| Watanabe et al. (2011) |  |  |  |  |  | *1* |  |  | *1* |  |  |  |
| Goto et al. (2015) |  |  |  |  |  | *1* |  |  |  |  |  |  |
| Dahlstrom et al. (1982) |  |  |  |  |  |  | *1* | *1* |  |  | *1* |  |
| Dahlstrom et al. (1984) |  |  |  |  |  |  | *1* | *1* |  |  |  |  |
| Dalen et al. (1986) |  |  |  |  |  |  | *1* |  |  |  | *1* |  |
| Dohrmann and Laskin (1978) |  |  |  |  |  |  | *1* |  |  |  |  |  |
| Hijzen et al. (1986) |  |  |  |  |  |  | *1* |  |  |  |  |  |
| Funch and Gale (1984) |  |  |  |  |  |  |  | *1* |  |  |  |  |
| Nishigawa et al. (2003) |  |  |  |  |  |  |  |  |  |  |  |  |
| Needham and Davies (2013) |  |  |  |  |  |  |  |  | *1* |  |  |  |
| Bergmann et al. (2020) |  |  |  |  |  |  |  |  |  | *1* |  |  |

**Table S5.** Biofeedback characteristics of included studies.

| *Treatment* | *Author(s), Year* | *Biofeedback intervention*  *Primary study* | *Comparison*  *Primary study* | *Type of Biofeedback*  *Primary study* | *Type of Diagnosis*  *SR* | *Age group*  *SR* | *Measurement site*  *Primary study* | *Treatment duration*  *Primary study* |
| --- | --- | --- | --- | --- | --- | --- | --- | --- |
| *Biofeedback* | *Aggarwal et al. (2019)* | *Tape recorded progressive muscle relaxation training program combined with EMG training (Crockett et al. 1986)*  *Biofeedback/Stress management (Turk et al. 1993/1996)*  *Biofeedback, Biofeedback & CBST (Gardea et al. 2001)*  *CBT & Biofeedback (Gatchel et al. 2006, Sheddan-Mora et al. 2013)* | *Other treatments (among them : no treatment, oral splint, cognitive therapy)* | *Auditory feedback (J&J Model M-51, J&J Model LGS-150 Digital Score Keeper) (Crockett et al. 1986)*  *Auditory & pulsating feedback (computer-controlled) (Turk et al. 1993)*  *Temperature & EMG feedback (AJ & J Model M-57 EMG, J & J Model T-68 Temperature Biofeedback Units) (Gardea et al. 2001)*  *Temperature & EMG feedback (Gatchel et al. 2006)*  *Multimodal feedback (10-channel FlexComp Infinity) (Sheddan-Mora et al. 2013)* | *TMD* | *Adults* | *Masseter muscle (bilaterally) (Crockett et al. 1986, Turk et al. 1993, Sheddan-Mora et al. 2013)*  *Frontales muscle (Gardea et al. 2001, Gatchel et al. 2006, Sheddan-Mora et al. 2013)*  *Temporalis muscle (Sheddan-Mora et al. 2013)*  *Trapezius muscle (Sheddan-Mora et al. 2013)* | *8 weeks (1 hour in-session and 30 min daily home exercises) (Crockett et al. 1986)*  *8 weeks (50 minutes in-session) (Sheddan-Mora et al. 2013)*  *6 weeks (1hour in-session) (Turk et al. 1993, Gatchel et al. 2006)*  *12 sessions (15 minutes) (Gardea et al. 2001)* |
|  | *Jokubauskas & Baltrušaitytė (2018)* | *CES (Jadidi et al. 2013, Raphael et al. 2013, Conti et al. 2014, Sumiya et al. 2014)*  *Biofeedback (Sato et al. 2014, Gu et al. 2015)* | *Other treatments (among them: placebo, no treatment)* | *CES (Grindcare) (Jadidi et al. 2013, Raphael et al. 2013, Conti et al. 2014)*  *CES (Portable EMG and ECG Telemetry System) (Sumiya et al. 2014)*  *Auditory feedback (One-Channel Portable EMG BF Device) (Sato et al. 2014)*  *Vibratory feedback (maxillary biofeedback splint) (Gu et al. 2015)* | *SB* | *Adults* | *Temporalis muscle (Jadidi et al. 2013, Raphael et al. 2013, Conti et al. 2014, Sato et al. 2014)*  *Masseter muscle (unilateral) (Sumiya et al. 2014)*  *Occlusal splint (Gu et al. 2015)* | *6 weeks (at least 5 nights per week) (Jadidi et al. 2013, Conti et al. 2014)*  *6 weeks (every night) (Raphael et al. 2013)*  *12 weeks (at night) (Gu et al. 2015)*  *2 days (Sato et al. 2014)*  *2 nights (Sumiya et al. 2014)* |
|  | *Miron et al. (2022)* | *Biofeedback (Bergmann et al. 2020)* | *Conventional occlusal splint* | *Biofeedback splint (bruXane splint) (Bergmann et al. 2020)* | *Nocturnal bruxism* | *Adults* | *Occlusal splint (Bergmann et al. 2020)* | *2 weeks (Bergmann et al. 2020)* |
|  | *Jedel & Carlsson (2003)* | *Biofeedback (Dahlstrom et al. 1982, Dalen et al. 1986)*  *Biofeedback, Biofeedback & CBST (Mishra et al. 2000)* | *Other treatments (among them : oral splint, control, cognitive therapy)* | *Temperature & EMG feedback (J&J Model M-57 EMG Biofeedback Unit and a J&J Model T-68 Temperature Biofeedback Unit) (Mishra et al. 2000)* | *TMD* | *Adults* | *Frontales muscles (Mishra et al. 2000)*  *Unclear (Masticatory muscles) (Dahlstrom et al. 1982, Dalen et al. 1986)* | *4 weeks (2 sessions per week) (Dalen et al. 1986)*  *6 weeks (Dahlstrom et al. 1982)*  *12 sessions (15 minutes) (Mishra et al. 2000)* |
| *Biofeedback* | *Wang et al. (2013)* | *Occlusal adjustment and biofeedback (Kardachi et al. 1978)*  *Nocturnal biofeedback (Casas et al. 1982)*  *Biofeedback (Pierce & Gale 1988, Wieselmann-Penkner et al. 2001)*  *CBT with partial nocturnal auditory biofeedback (Ommerborn et al. 2007)*  *CES (Jadidi et al. 2008, Jadidi et al. 2011)* | *Other treatments (among them: placebo, stress-reduction behavioral counseling, no treatment, oral splint, massed negative practice, TENS)* | *Auditory feedback (portable EMG unit) (Kardachi et al. 1978, Casas et al. 1982, Pierce & Gale 1988)*  *Auditory feedback (Bruxcore bruxism-monitoring device) (Ommerborn et al. 2007)*  *Diurnal relaxation (Pierce & Gale 1988)*  *CES (Grindcare) (Jadidi et al. 2008, Jadidi et al. 2011)*  *Visual feedback (computer aided biofeedback system) (Wieselmann-Penkner et al. 2001)* | *SB* | *Adults* | *Masseter muscle (Kardachi et al. 1978, Casas et al. 1982, Pierce & Gale 1988, Wieselmann-Penkner et al. 2001, Ommerborn et al. 2007)*  *Temporalis muscle (Kardachi et al. 1978, Wieselmann-Penkner et al. 2001, Jadidi et al. 2007, Jadidi et al. 2011)*  *Nasion (Wieselmann-Penkner et al.2001)* | *1 night (Jadidi et al. 2011)*  *1 week (Kardachi et al. 1978)*  *2 weeks (at night) (Casas et al. 1982, Pierce & Gale 1988, Ommerborn et al. 2007)*  *6 weeks (at least 5 nights per week) (Jadidi et al. 2008)*  *10 1-minute trial during each of 6 treatment sessions (Pierce & Gale 1988)*  *Weekly 3 times (20 minutes for one session) (Wieselmann-Penkner et al. 2001)* |
|  | *Yao et al. (2023)* | *Biofeedback/Stress management (Turk et al. 1993)*  *CBT & Biofeedback (Sheddan-Mora et al. 2013)*  *Biofeedback, Biofeedback & CBST (Mishra et al. 2000)*  *Biofeedback (Barbosa et al. 2019)* | *Other treatments (among them: no treatment, oral splint, cognitive therapy, placebo)* | *Auditory & pulsating feedback (computer-controlled) (Turk et al. 1993)*  *Multimodal feedback (10-channel FlexComp Infinity) (Sheddan-Mora et al. 2013)*  *Temperature & EMG feedback (J&J Model M-57 EMG Biofeedback Unit and a J&J Model T-68 Temperature Biofeedback Unit) (Mishra et al. 2000)*  *Visual feedback (BiotrainerTM visual biofeedback software) (Barbosa et al. 2019)* | *Chronic pain associated with TMD* | *Adults* | *Masseter muscle (bilaterally) (Turk et al. 1993, Sheddan-Mora et al. 2013, Barbosa et al. 2019)*  *Frontales muscles (Mishra et al. 2000, Barbosa et al. 2019)* | *6 weeks (1hour in-session) (Turk et al. 1993)*  *8 weeks (50 minutes in-session) (Sheddan-Mora et al. 2013)*  *8 weeks (16 sessions) (Barbosa et al. 2019)*  *12 sessions (15 minutes) (Mishra et al. 2000)* |
| *Biofeedback* | *Florjanski et al. (2019)* | *Biofeedback (Wieselmann-Penker et al. 2001, Gu et al. 2015, Criado et al. 2016, Watanabe et al. 2011, Sato et al. 2015, Goto et al. 2015)*  *CES (Conti et al. 2014, Raphael et al. 2013, Jadidi et al. 2008, Sumiya et al. 2014)* | *Other treatments (among them : TENS, oral splint, control)* | *Visual feedback (Wieselmann-Penker et al. 2001, Criado et al. 2016)*  *Auditory feedback (Watanabe et al. 2011, Sato et al. 2015, Goto et al. 2015)*  *Vibratory feedback (Gu et al. 2015)*  *CES (Conti et al. 2014, Raphael et al. 2013, Jadidi et al. 2008, Sumiya et al. 2014)* | *TMD* | *Adults* | *Masseter muscle (Wieselmann-Penker et al. 2001, Criado et al. 2006, Goto et al. 2015, Sumiya et al. 2014)*  *Temporalis muscle (Wieselmann-Penker et al. 2001, Criado et al. 2006, Watanabe et al. 2011, Sato et al. 2015, Conti et al. 2014, Raphael et al. 2013, Jadidi et al. 2008)*  *No electrodes, pressure sensor in the device (Gu et al. 2015)* | *2 days (Watanabe et al. 2011, Sato et al. 2015)*  *2 consecutive nights (Sumiya et al. 2014)*  *3 consecutive nights (Goto et al. 2015)*  *At least 10 days (Conti et al.2014)*  *2 weeks (2 sessions per week) (Criado et al. 2016)*  *3 weeks (1 session per week, 20 minutes) (Wieselmann-Penker et al. 2001)*  *6 weeks (every night) (Raphael et al. 2013)*  *6 weeks (at least 5 nights per week) (Jadidi et al. 2008)*  *12 weeks (Gu et al. 2015)* |
|  | *Iwasaki et al. (2018)* | *CBT & Biofeedback (Gatchel et al. 2006)* | *No intervention* | *Temperature & EMG feedback (Gatchel et al. 2006)* | *TMD* | *Adults* | *Frontales muscle (Gatchel et al. 2006)* | *6 weeks (1 session per week) (Gatchel et al. 2006* |
|  | *McNeely et al. (2006)* | *Biofeedback (Wieselmann-Penkner et al. 2001, Dahlstrom et al. 1982, Dahlstrom et al.1984, Funch & Gale 1984)* | *Other treatments (among them: relaxation, oral splint, TENS, control)* | *Visual feedback (computer aided biofeedback system) (Wieselmann-Penkner et al. 2001)*  *EMG feedback (Dahlstrom et al. 1982, Dahlstrom et al.1984)*  *Unclear (Funch & Gale 1984)* | *TMD* | *Adults* | *Masseter muscle (Wieselmann-Penkner et al. 2001, Dahlstrom et al.1984)*  *Temporalis muscle (Wieselmann-Penkner et al. 2001)*  *Frontalis muscle (Dahlstrom et al.1984)*  *Nasion (Wieselmann-Penkner et al. 2001)*  *Unclear (Masticatory muscles) (Dahlstrom et al. 1982, Funch & Gale 1984)* | *3 sessions (Funch & Gale 1984)*  *Weekly 3 times (20 minutes for one session) (Wieselmann-Penkner et al. 2001)*  *6 weeks (Dahlstrom et al. 1982, Dahlstrom et al.1984)* |
| *Biofeedback* | *Amorim et al. (2018)* | *CES (Raphael et al. 2013, Conti et al. 2014, Needham and Davies 2013, Jadidi et al. 2008/2011/2013, Sumiya et al. 2014)*  *Biofeedback (Nishigawa et al. 2003, Wieselmann-Penkner et al. 2001, Watanabe et al. 2011, Gu et al. 2015, Sato et al. 2015)*  *CBT with partial nocturnal auditory biofeedback (Ommerborn et al. 2007)* | *Other treatments (among them: TENS, no treatment, control, oral splint)* | *CES (Raphael et al. 2013, Conti et al.2014, Needham and Davies 2013, Jadidi et al. 2008/2011/2013, Sumiya et al. 2014)*  *Labial feedback (contingent electrical lip stimulation) (Nishigawa et al. 2003)*  *Visual feedback (computer aided biofeedback system) (Wieselmann-Penkner et al. 2001)*  *Auditory feedback (Watanabe et al. 2011, Ommerborn et al. 2007, Sato et al. 2015)*  *Vibratory feedback (Gu et al. 2015)* | *AB and SB* | *Adults* | *Frontales muscle (Raphael et al. 2013)*  *Temporalis muscle (Wieselmann-Penkner et al. 2001, Watanabe et al. 2011, Conti et al.2014, Needham and Davies 2013, Jadidi et al. 2008/2011/2013, Sato et al. 2015)*  *Masseter muscle (Nishigawa et al. 2003, Wieselmann-Penkner et al. 2001, Ommerborn et al. 2007, Sumiya et al. 2014)*  *Nasion (Wieselmann-Penkner et al. 2001)*  *No electrodes, pressure sensor in the device (Gu et al. 2015)* | *1 night (Jadidi et al. 2011)*  *2 days (Watanabe et al. 2011)*  *2 consecutive nights (Sumiya et al. 2014)*  *3 consecutive nights (Goto et al. 2015)*  *5 nights (delivered in 1-hour blocks for half of each sleep period) (Nishigawa et al. 2003)*  *At least 10 days (Conti et al.2014)*  *2 weeks (at night) (Ommerborn et al. 2007)*  *5 weeks (every night) (Needham and Davies 2013)*  *6 weeks (every night) (Raphael et al. 2013)*  *6 weeks (at least 5 nights per week) (Jadidi et al. 2008/2013)*  *Weekly 3 times (20 minutes for one session) (Wieselmann-Penkner et al. 2001)*  *12 weeks (Gu et al. 2015)* |
| *Biofeedback* | *Medlicott & Harris (2006)* | *Tape recorded progressive muscle relaxation training program combined with EMG training (Crockett et al. 1986)*  *Biofeedback (Dahlstrom et al. 1982, Dahlstrom and Carlson 1984, Dalen et al. 1986, Dohrmann and Laskin 1978, Hijzen et al. 1986)*  *Biofeedback/Stress management (Turk et al. 1993)* | *Other treatments* | *Auditory feedback (J&J Model M-51, J&J Model LGS-150 Digital Score Keeper) (Crockett et al. 1986)*  *EMG feedback (Dahlstrom et al. 1982, Dahlstrom et al.1984, Dalen et al. 1986, Dohrmann and Laskin 1978)*  *Auditory & pulsating feedback (computer-controlled) (Turk et al. 1993)* | *TMD* | *Adults* | *Masseter muscle (bilaterally) (Crockett et al. 1986, Turk et al. 1993)*  *Unclear (Masticatory muscles) (Dahlstrom et al. 1982, Dahlstrom et al.1984, Dalen et al. 1986, Dohrmann and Laskin 1978)* | *4 weeks (2 sessions per week)(Dalen et al. 1986)*  *5 weeks (10 times) (Hijzen et al. 1986)*  *6 weeks (1hour in-session) (Turk et al. 1993)*  *6 weeks (Dahlstrom et al. 1982, Dahlstrom and Carlson 1984)*  *6 weeks (2 sessions per week) (Dohrmann and Laskin 1978)*  *8 weeks (1 hour in-session and 30 min daily home exercises) (Crockett et al. 1986)* |

*CBST (cognitive-behavioral skills training), CBT (cognitive-behavioral therapy), CED (contingent electrical stimulation), BF (biofeedback), SB (sleep bruxism), AB (awake bruxism)*
